# Elimination of damaged mitochondria during UVB‐induced senescence is orchestrated by NIX‐dependent mitophagy

**DOI:** 10.1111/acel.14186

**Published:** 2024-05-17

**Authors:** Maria Cavinato, Ines Martic, Sophia Wedel, Annabella Pittl, Rafal Koziel, Regina Weinmmüllner, Markus Schosserer, Brigitte Jenewein, Madhusudhan Reddy Bobbili, Elsa Arcalis, Johannes Haybaeck, Gerhard Pierer, Christian Ploner, Martin Hermann, Nikolaus Romani, Matthias Schmuth, Johannes Grillari, Pidder Jansen‐Dürr

**Affiliations:** ^1^ Institute for Biomedical Aging Research University of Innsbruck Innsbruck Austria; ^2^ Center for Molecular Biosciences Innsbruck (CMBI) Innsbruck Austria; ^3^ Institute of Molecular Biotechnology University of Natural Resources and Life Sciences Vienna Austria; ^4^ Institute of Medical Genetics, Center for Pathobiochemistry and Genetics Medical University Vienna Vienna Austria; ^5^ Austrian Cluster for Tissue Regeneration Vienna Austria; ^6^ Ludwig Boltzmann Institute for Traumatology, The Research Center in Cooperation with AUVA Vienna Austria; ^7^ Institut für Pflanzenbiotechnologie und Zellbiologie University of Natural Resources and Life Sciences (BOKU) Vienna Austria; ^8^ Institute of Pathology, Neuropathology and Molecular Pathology Medical University of Innsbruck Innsbruck Austria; ^9^ Department of Pathology Saint Vincent Hospital Zams Zams Austria; ^10^ Department of Pathology, Labor Team Goldach Switzerland; ^11^ Department of Plastic, Reconstructive and Aesthetic Surgery Medical University of Innsbruck Innsbruck Austria; ^12^ Department of Anesthesiology and Critical Care Medicine Medical University of Innsbruck Innsbruck Austria; ^13^ Department of Dermatology, Venereology and Allergology Medical University of Innsbruck Innsbruck Austria; ^14^ Present address: Department of Internal Medicin V, Hematology & Oncology Tirol Kliniken Innsbruck Innsbruck Austria; ^15^ Present address: Biosens Labs Ltd. Warsaw Poland

**Keywords:** mitochondria, mitophagy, NIX, senescence, skin aging, UVB, vesicles

## Abstract

Skin aging is the result of two types of aging, “intrinsic aging” an inevitable consequence of physiologic and genetically determined changes and “extrinsic aging,” which is dependent on external factors such as exposure to sunlight, smoking, and dietary habits. UVB causes skin injury through the generation of free radicals and other oxidative byproducts, also contributing to DNA damage. Appearance and accumulation of senescent cells in the skin are considered one of the hallmarks of aging in this tissue. Mitochondria play an important role for the development of cellular senescence, in particular stress‐induced senescence of human cells. However, many aspects of mitochondrial physiology relevant to cellular senescence and extrinsic skin aging remain to be unraveled. Here, we demonstrate that mitochondria damaged by UVB irradiation of human dermal fibroblasts (HDF) are eliminated by NIX‐dependent mitophagy and that this process is important for cell survival under these conditions. Additionally, UVB‐irradiation of human dermal fibroblasts (HDF) induces the shedding of extracellular vesicles (EVs), and this process is significantly enhanced in UVB‐irradiated NIX‐depleted cells. Our findings establish NIX as the main mitophagy receptor in the process of UVB‐induced senescence and suggest the release of EVs as an alternative mechanism of mitochondrial quality control in HDF.

## Graphical abstract

Upon sporadic UVB exposure (1), NIX is translocated from the nucleus to damaged mitochondria, inducing NIX‐dependent mitophagy (NDM). As a result, cells can recycle their mitochondria and remain healthy. Under chronic UVB exposure (2), NDM is not sufficient to fully restore mitochondrial function, resulting in cellular senescence. Upon inhibition of NDM (3), cells release EVs containing mitochondria. Under these conditions, a shift from senescence to cell death is observed.

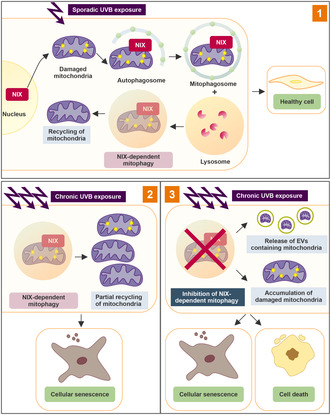

**Abbreviations**
CV: Complex V
DMEM: Dulbecco's modified eagle medium
EVs: Extracellular vesicles
FACS: Fluorescence activated cell sorting
GFP: Green fluorescent protein
HBSS: Hank's balanced salt solution
HDF: Human dermal fibroblasts
LTB: Lysotracker blue
MDVs: Mitochondria‐derived vesicles
MTDR: Mitotracker deep red
MTG: Mitotracker green
NTA: Nanoparticle tracking analysis
q‐RT‐PCR: Quantitative real‐time polymerase chain reaction
ROS: Reactive oxygen species
SA‐β‐Gal: Senescence‐associated beta galactosidase
Scr: Scrambled
SEC: Size‐exclusion chromatography
shRNA: Small hairpin RNA
TEM: Transmission electron microscopy
UVB: Ultraviolet B
WB: Western blot

## 1 INTRODUCTION

Skin aging results in the reduction of the normal and mechanical protective function of the skin with a significant impact on the quality of life (Blume‐Peytavi et al., 2016). In addition, skin is a social organ, and alterations in appearance of this tissue can cause psychosocial distress and affect an individual's social behavior and well‐being (Cavinato, 2020). Molecular processes leading to aging of the skin are incompletely understood, and the identification of mechanisms contributing to this condition will provide new targets for intervention that are urgently needed.

Chronic exposure to sunlight is associated with cumulative damage to the skin and impairment of skin function raising the risk of long‐term consequences, such as photoaging and photocarcinogenesis (Bosch et al., 2015). UVB irradiation (280–320 nm) is harmful to biological tissues since it can directly damage molecules such as nucleic acids and proteins (Lorencini et al., 2014). At a molecular level, UVB likewise interacts with cellular chromophores resulting in DNA damage and generation of reactive oxygen species (ROS), in turn leading to the activation of multiple cell signaling pathways related to cell growth, senescence, DNA damage repair, mitochondrial physiology and mechanisms of protein quality control (Cavinato & Jansen‐Dürr, 2017).

In the skin, senescent cells accumulate both in the dermis and in the epidermis during aging (Velarde & Demaria, 2016; Victorelli et al., 2019; Wang et al., 2017; Wang & Dreesen, 2018) affecting not only the functions of the skin layers but also the tissue as a whole, and contribute to the characteristic functional decline of the aging process (Campisi, 1998). Mitochondrial dysfunction plays an important role in the development of cellular senescence, in particular stress‐induced senescence of human cells (Correia‐Melo et al., 2016; Gallage & Gil, 2016; Wiley et al., 2016; Ziegler et al., 2015). Mitophagy, a selective form of macro‐autophagy in which mitochondria are specifically targeted for autophagic degradation (Quiles & Gustafsson, 2020), regulates mitochondrial turnover and is essential for cellular homeostasis by recycling mitochondrial contents and macromolecules (De Gaetano et al., 2021; Li et al., 2021). Accumulation of dysfunctional or superfluous mitochondria as well as impairment of mitochondrial biogenesis due to decreased functionality of mitophagy are major characteristics of several human pathophysiological and age‐related conditions, highlighting the pivotal role of mitochondrial quality control in healthspan maintenance and longevity (Lionaki et al., 2015; Palikaras et al., 2015; Von Stockum et al., 2016).

Cells perform mitophagy through several non‐redundant and partially tissue‐specific mechanisms. Specifically in mammalian cells, there are several ways by which damaged mitochondria are recognized and engulfed by the autophagic machinery (Yoo & Jung, 2018). Some of these mechanisms depend on the ubiquitination of mitochondrial proteins followed by interaction with the adaptor proteins connecting ubiquitin with LC3 (Lazarou et al., 2015; Moore & Holzbaur, 2016). Other mitophagy mechanisms use proteins on the mitochondrial membrane as direct receptors for LC3 (Saito & Sadoshima, 2015). For each of these processes, mitophagy receptors and related proteins have been described (Ding et al., 2010; Liu et al., 2014; Wei et al., 2017; Zhang et al., 2017). BCL2/adenovirus E1B 19 kDa protein‐interacting protein 3‐like, BNIP3L/NIX, an outer mitochondrial membrane protein is crucial for the elimination of mitochondria during red blood cell maturation (Sandoval et al., 2008; Schweers et al., 2007). NIX also participates in the promotion of mitophagy upon mitochondrial depolarization, hypoxia/ischemia, and excessive reactive oxygen species generation (Ding et al., 2010; Lampert et al., 2019). Recently, NIX‐dependent mitophagy has been described as one of the key mechanisms involved in the differentiation of epidermal keratinocytes, participating in the physiological elimination of unneeded mitochondria in the upper epidermal layers (Simpson et al., 2021). The role of NIX‐dependent mitophagy in the dermis is, however, not yet elucidated.

In addition to mitophagy, other mechanisms of mitochondrial quality control safeguard mitochondrial and cellular homeostasis. Among them are the eviction of mitochondrial components via the generation and release of mitochondrial‐derived vesicles (MDVs) (Cadete et al., 2016; Soubannier, Rippstein, et al., 2012; Todkar et al., 2021). In fact, several studies have identified the elimination of mitochondrial proteins as extracellular vesicles (EVs) cargo in response to stress (Cadete et al., 2016; Nicolás‐Ávila et al., 2020; Todkar et al., 2021; Vasam et al., 2021) and suggest that budding of MDVs might work as an alternative mechanism to compensate for deficient mitophagy (Phinney et al., 2015) independent of mitochondrial depolarization, autophagy signaling, or mitochondrial fission. Moreover, the presence of mitochondrial cargo within EV or the release of free mitochondria in the extracellular space were shown to induce the production of pro‐inflammatory factors (Todkar et al., 2021; Zhang et al., 2010) and might be ultimately related to the aging process (Picca et al., 2019, 2020).

Elimination of mitochondria by mitophagy during stress conditions in different systems has been well‐documented in the last years (Schofield & Schafer, 2020; Swiader et al., 2016; Yang et al., 2020). However, many aspects of mitochondrial physiology and quality control relevant to the process of skin homeostasis and photoaging remain to be unraveled. Here, we have explored mechanisms of mitochondrial quality control in the process of UVB‐induced senescence of fibroblasts and have demonstrated that NIX‐dependent mitophagy is the main mechanism of elimination of damaged mitochondria upon UVB irradiation. Additionally, we have shown that release of EVs containing mitochondria is a possible alternative mechanism of mitochondrial quality control that can complement mitophagy in conditions of stress and can compensate, at least in part, for mitophagy impairment in the absence of NIX.

## 2 RESULTS

### 2.1 UVB impairs mitochondrial physiology and morphology

**FIGURE 1:**
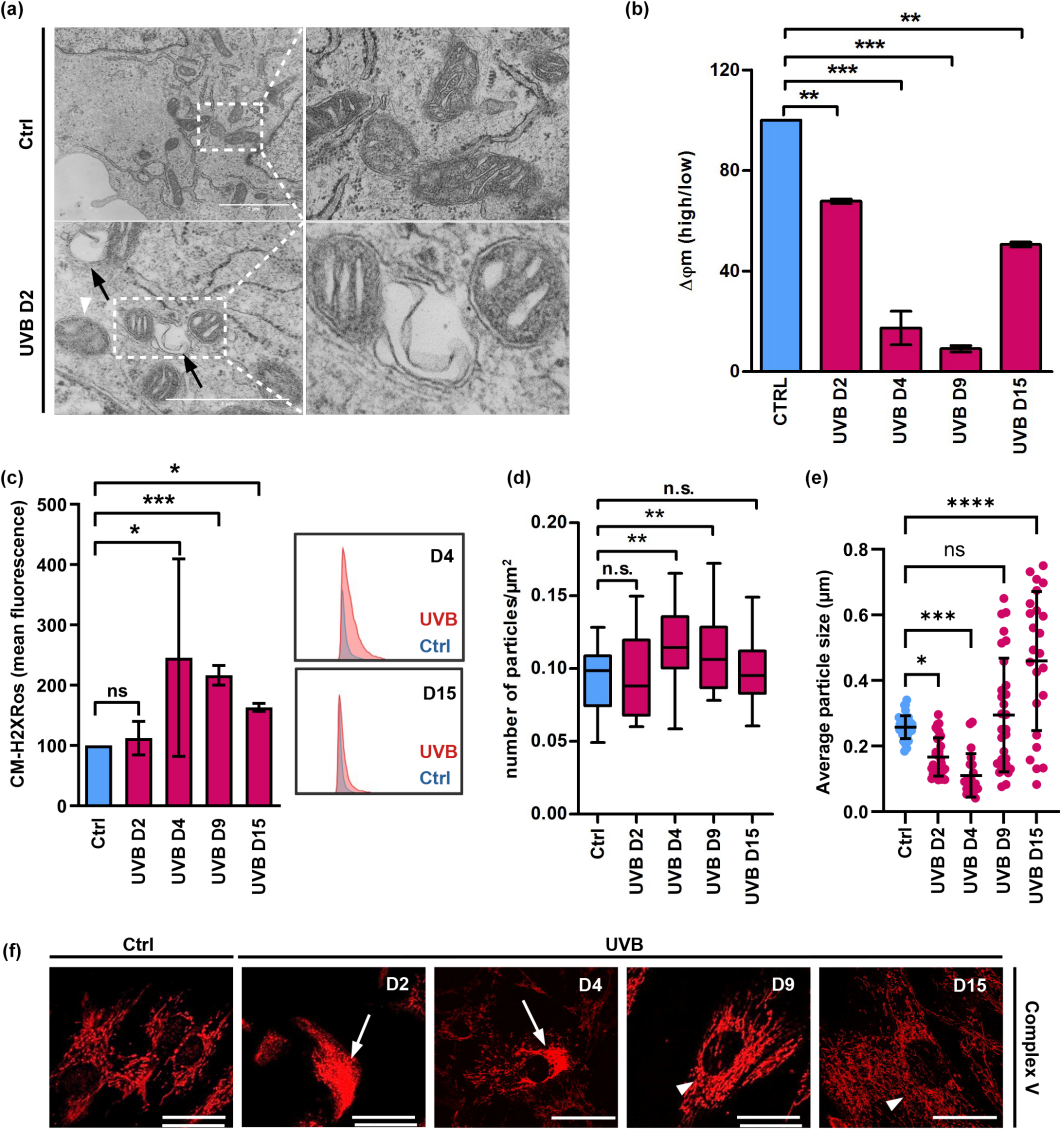
UVB irradiation impairs mitochondrial morphology and physiology. (a) Human dermal fibroblasts submitted to 2 days of UVB irradiation, and the corresponding controls were processed and analyzed by TEM. Details show healthy mitochondria in control non‐irradiated cells and blebbing damaged mitochondria after 2 days of UVB irradiation. *Black arrows*: blebbing of mitochondrial membrane, *white arrowheads*: mitochondria with disorganized cristae. (b, c) UVB‐irradiated and control HDF were labeled with the fluorescent probes JC‐1 (b) and CM‐H_2_XRos (c) and analyzed by FACS for the evaluation of the effects of UVB on mitochondrial membrane potential and mitochondrial ROS levels, respectively (b) Mitochondrial membrane potential of Ctrl and UVB‐irradiated cells. Bars represent the ratio obtained by dividing the percentage of mitochondria with high membrane potential by the percentage of mitochondria with low membrane potential of a given population. Results are displayed as mean values of three independent biological samples ± SD. Controls were normalized to 100% for better interpretation of the results. (c) Mitochondrial ROS in Ctrl and UVB‐irradiated HDF. Results are displayed as mean values ± SD of three independent experiments. Histograms are representative of control (blue) and irradiated (red) cells measured on days 4 and 15 of the experiment. (d–f) Human dermal fibroblasts were irradiated, labeled by indirect immunofluorescence with antibody to detect the mitochondrial Complex V subunit ATP Synthase beta and observed in confocal microscopy to evaluate mitochondrial network morphology. (d, e) Analysis of mitochondrial network fragmentation (d) and mitochondrial particle size (e) was performed in ImageJ software using the images obtained from 90 cells randomly chosen from three independent experiments. (f) Representative images of mitochondria in control and UVB‐irradiated HDF. *White arrows*: impaired mitochondrial network. *White arrowheads*: recovered mitochondrial network. Scale bar: 10 μm. For all experiments: *p ≤ 0.05; **p ≤ 0.01; ***p ≤ 0.001; n.s., non‐significant.

In order to investigate the effects of UVB‐induced senescence on mitochondrial morphology and function, we used a pre‐established protocol of UVB irradiation of HDFs (Cavinato et al., 2016; Greussing et al., 2013). Cells were subjected to UVB irradiation twice daily for four consecutive days (D1 to D4), resulting in a cumulative dose corresponding to 16,000 standard erythemal doses (SED) (Salvadori et al., 2019), equivalent to approximately 25 years of constant sun exposure in a tropical region (Mendes et al., 2020). Following irradiation, cells were maintained in culture for an additional 11 days (D5 to D15) to monitor the development of the senescent phenotype over time. Ultrastructural analysis by transmission electron microscopy (TEM) revealed massive morphological disruption of mitochondria by UVB irradiation. Mitochondria of control cells varied in size and shape, were delimited by a double membrane, and displayed numerous clearly discernible cristae (Figure 1a, Ctrl detail). In UVB‐treated cells, both normal and aberrant mitochondria were observed. Aberrant mitochondria were characterized by blebbing of the outer membrane (Figure 1a, UVB D2 detail) and by disorganization of the cristae (Figure 1a, white arrowheads). Mitochondrial membrane potential decreased drastically in UVB‐irradiated cells starting from Day 2 after the first irradiation (D2) showing a partial recovery at the end of the experiment (D15) (Figure 1b). As expected, we observed that UVB‐irradiated cells presented increased mitochondrial ROS levels in comparison with control non‐irradiated cells (Figure 1c). Accordingly, mitochondrial morphology was also affected by UVB as revealed by indirect immunofluorescence analysis to label the mitochondrial Complex V subunit ATP Synthase beta (hereafter named Complex V). In control cells of all groups, mitochondria formed large, interconnected networks, uniformly distributed throughout the cytoplasm with few fragmented regions (Figure 1d–f). In contrast, UVB caused disruption of the network, already after the first doses, leading to the appearance of smaller and fragmented mitochondria unevenly spread in the cytoplasm. This pattern was especially prominent on D4, when the maximum number of smaller mitochondrial fragments per cell was observed. However, starting from D9, fragmented mitochondria of irradiated cells were replaced by mitochondria with regular morphology (Figure 1d–f). Altogether these results demonstrate that sub‐cytotoxic and repeated doses of UVB induce transient morphological and functional damage to mitochondria.

### 2.2 Damaged mitochondria generated by UVB irradiation are eliminated by mitophagy in HDF

**FIGURE 2:**
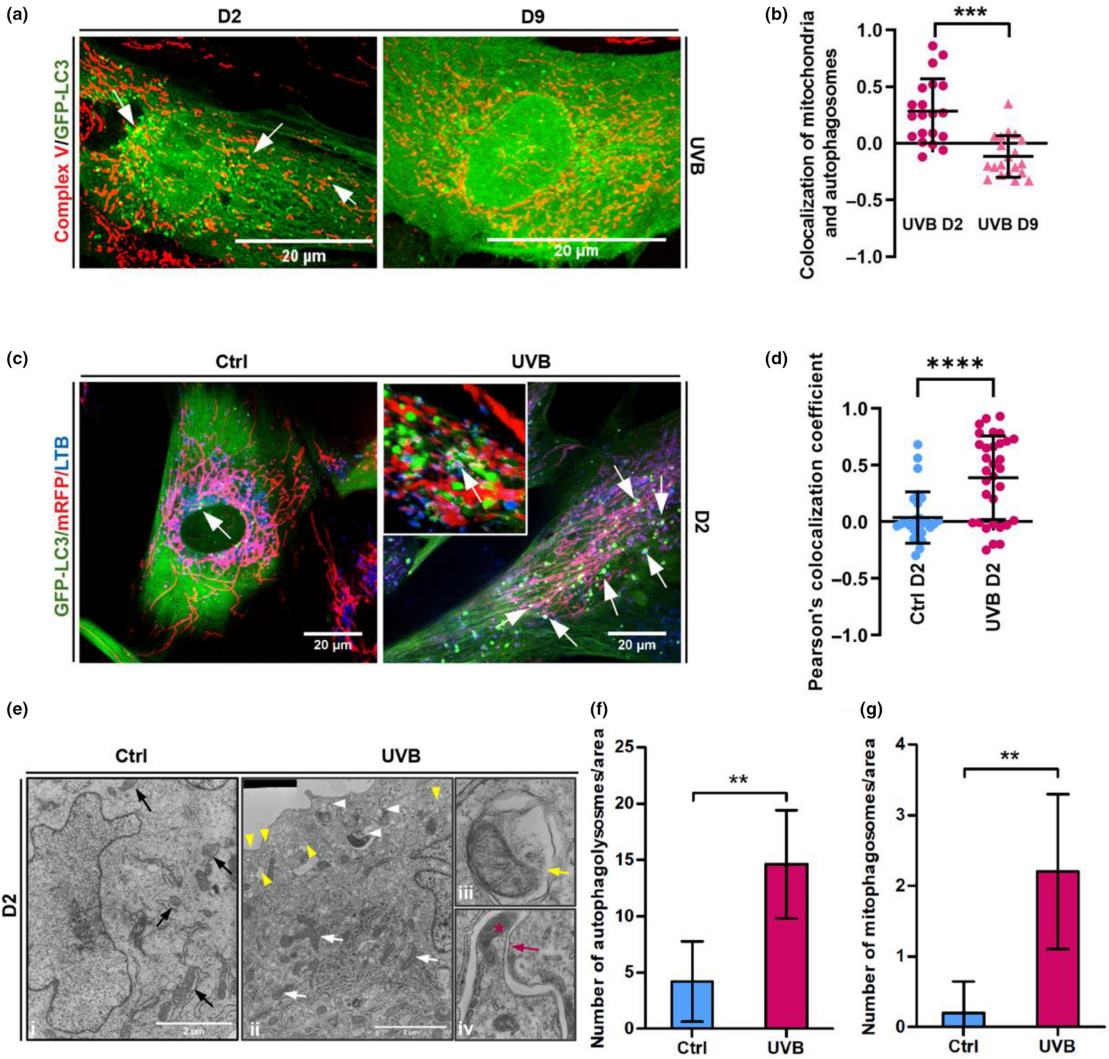
Mitochondria damaged by UVB are eliminated by mitophagy. (a) HDF expressing GFP‐LC3 were grown on coverslips and UVB‐irradiated. Immediately after the second day of irradiation (D2) or after 4 days of irradiation followed by 5 days of recovery (D9), cells were fixed and stained by indirect immunofluorescence with anti‐complex V (red) antibody and observed by confocal microscope. Single channels for each figure are displayed in Figure S1A. Colocalization of mitochondria and autophagosomes (*white arrows*) was quantified in ImageJ software and coefficient of colocalization of at least 30 cells of three independent experiments is represented in (b). Values above 0 represent positive colocalization and values under 0 represent negative colocalization. (c) GFP‐LC3/mRFP‐expressing fibroblasts exposed to 2 days of irradiation and the corresponding controls were stained with Lysotracker® Blue and observed by live cell confocal microscopy to investigate the elimination of mitophagosomes by mitophagy. *White arrows* point the co‐localization of autophagosomes (green), mitochondria (red) and lysosomes (blue). Images are representative of three independent experiments. Inset shows 3D detailed colocalization of mitochondria, autophagosomes and lysosomes in a UVB‐irradiated cells. Scale bars: 20 μm. Single channels for each picture are displayed in Figure S1F. (d) Coefficient of colocalization of mitochondria and lysosomes of control and UVB‐irradiated cells obtained by ImageJ software. Results are represented as mean value ± SD of three independent experiments. (e) Human dermal fibroblasts submitted to 2 days of UVB (ii‐iv) and the corresponding controls (i) were processed and analyzed by TEM to confirm the occurrence of mitophagy upon UVB irradiation. *Black arrows* (*i*): healthy mitochondria observed in control cells. *White arrows* (*ii*): mitochondria with damaged cristae in UVB‐irradiated cells. *White arrowheads* (*ii*): damaged mitochondria being engulfed by a forming autophagosome. *Yellow arrowheads* (*ii*): non‐mitophagic vesicles. *iii*: mitochondria contained inside mitophagosome (*yellow arrow*) in UVB‐treated HDFs. *iv*: mitochondria (red star) being engulfed by a forming autophagosome (red arrow). Scale bar: 2 μm. (f, g) Quantification of number of autophagy‐related organelles (f) and mitophagosomes (g) observed in electromicrographs from Control and UVB‐irradiated cells. Bar graphs represent the mean values ± SD of at least 10 cells. For all experiments: *p ≤ 0.05; **p ≤ 0.01; ***p ≤ 0.001; n.s., non‐significant.

Compromised mitochondrial integrity has serious consequences in mammalian cells and is linked to senescence, aging, and degenerative diseases in humans (López‐Otín et al., 2013; Wiley et al., 2016). As an adaptive response to mitotoxic stress, several mechanisms that act at different levels and times have been evolutionarily developed to guarantee high quality of these organelles. Partial recovery of mitochondrial structure and function following UVB irradiation of HDF suggested that a mechanism(s) of mitochondrial quality control must be active during and after exposure to this type of stress. Several mitochondrial quality control pathways including the cytosolic ubiquitin‐proteasome system, mitophagy, and the generation of MDVs are described to maintain the normal physiological state of cells (Picca et al., 2020). Mitophagy stands out as the main mechanism for elimination of damaged mitochondria in eukaryotes and plays a role in protecting epidermal keratinocytes from UVB‐induced damage (Moriyama et al., 2017). To investigate the occurrence and possible role of mitophagy in UVB‐treated HDF, we employed HDF lines overexpressing the autophagy‐related protein LC3 fused, at its N terminus, to the green fluorescent protein (GFP). GFP‐LC3‐expressing cells were exposed to UVB and processed for immunofluorescence to label Complex V. Control cells exhibited diffuse LC3 fluorescence in the cytoplasm, with few autophagosomes (Figure S1A) while UVB‐irradiated cells displayed an increased number of autophagosomes during the period of irradiations (UVB D2) followed by subsequent recovery at D9 (Figure 2a and Figure S1A). The transient induction of autophagy in the first days of the experiment coincided with the increased colocalization between mitochondria and autophagosomes, as measured by colocalization of LC3 positive puncta and Complex V (Figure 2b), as well as by live‐cell microscopy using the GFP‐LC3/mRFP double reporter cell line (Video S1). Interestingly, regions of colocalization between mitochondria and autophagosomes decreased after the period of irradiations and were strongly reduced at day 9 (Figure 2a,b and Figure S1A), when the majority of the cells reached senescence (Figure 1b–e). Mitophagic flux was monitored by staining the cells with LysoTracker® Blue (LTB) immediately after irradiation and prior to observation by confocal microscopy. LTB is a fluorescent dye that stains acidic compartments in living cells and provides a useful tool to monitor, in real‐time, the progress of autophagy subsequent to the formation of autophagosomes. UVB irradiation increased the colocalization of mitochondria, autophagosomes, and lysosomes in comparison with control non‐irradiated cells (Figure 2c,d, Figure S1F). Notably, when treating cells with the autophagy inhibitor Bafilomycin A (Figure S2G), we confirmed that mitophagic flux is increased by UVB irradiation. Occurrence of mitophagy upon UVB irradiation was further investigated by electron microscopy (Figure 2e–g). Consistent with results obtained by confocal microscopy, electron micrographs confirmed an increased number of autophagolysosomes as well as the appearance of mitochondria engulfed by autophagosomes in UVB‐irradiated HDFs. Interestingly, mitophagosomes were mainly localized in the periphery of the cells (Figure 2e, white arrowheads), where we also observed the accumulation of non‐mitophagic vesicles (Figure 2e, yellow arrowheads). Taken together, these results demonstrate that massive mitochondrial damage caused by UVB irradiation is cleared, at least partially, by mitophagy in HDF.

### 2.3 UVB irradiation increases expression of NIX and triggers its translocation from the nucleus to the mitochondria

Distinct mechanisms of mitophagy are activated by different mitochondrial stresses. Still, in all cases, target mitochondria are recognized by autophagosomes in a process that can be either mediated by LC3 adapters, which function in a ubiquitin‐dependent or independent manner, or through the direct interaction of LC3 with its receptors. In mammals, several mitophagy effectors such as the mitophagy receptors NIX, BNIP3, and FUNDC1 as well as the PINK1/PARKIN pathway were found to participate in the selective clearance of mitochondria (Wei et al., 2015; Yoo & Jung, 2018).

**FIGURE 3:**
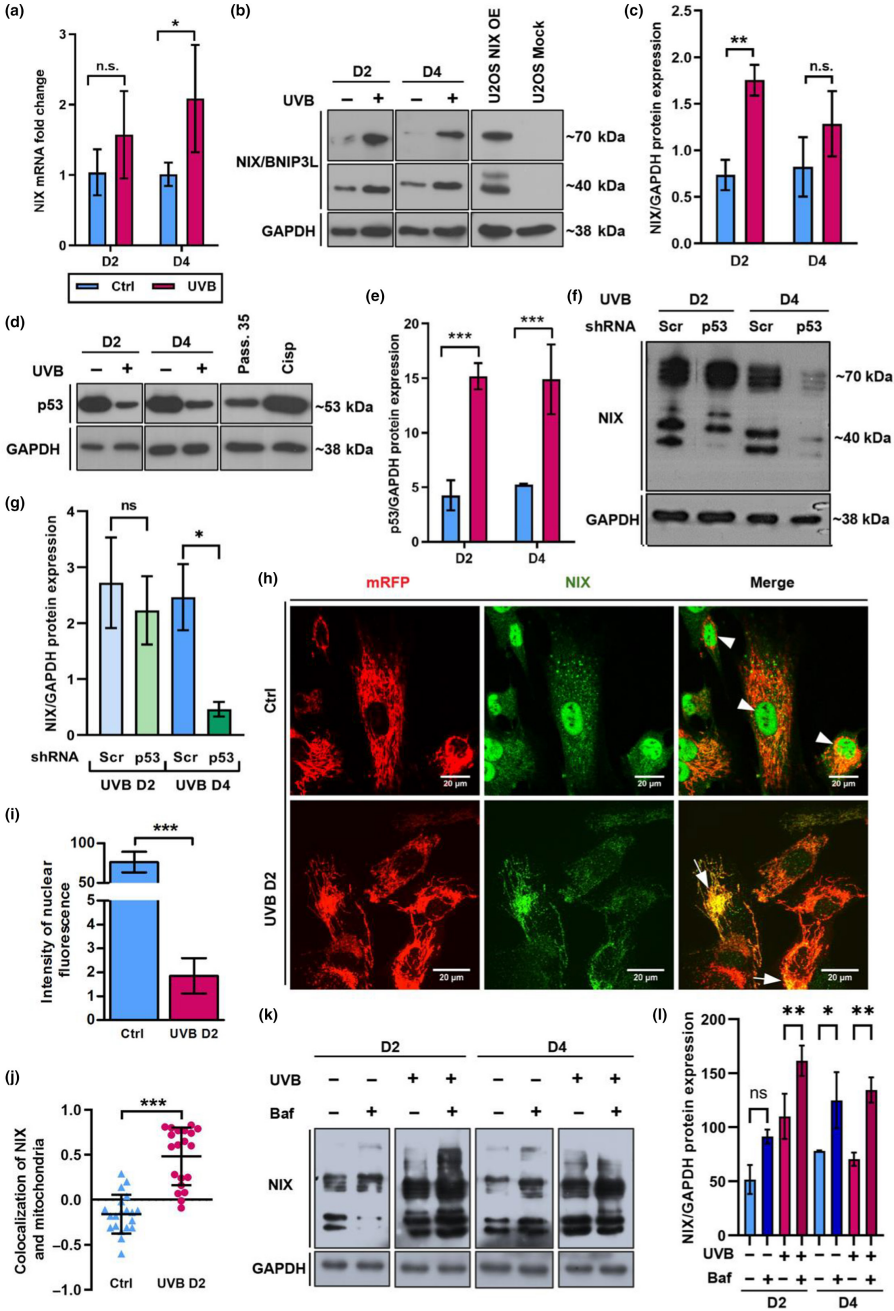
Expression and subcellular localization of mitophagy receptor NIX are regulated during UVB‐induced senescence. (a, b) Wild type HDF were irradiated as described. mRNA samples and protein lysates were obtained and analyzed by q‐RT‐PCR (a) and WB (b) respectively, to verify regulation of the mitophagy receptor NIX upon UVB. (a) Bars represent mean values of three independent experiments ± SD. (b) NIX protein expression was analyzed by WB in UVB‐irradiated and control HDFs. Lysates of U2OS transfected with NIX overexpression and Mock (empty) plasmids were used as positive and negative controls for the antibody specificity, respectively. Images are representative of three independent biological replicates. (c) Bar graph represents mean values obtained by densitometry evaluation of NIX positive bands of three independent experiments ± SD. (d) WB to evaluate the expression of upstream NIX regulator p53 in control and UVB‐irradiated cells. HDF at passage 35 (Pass. 35) and HDF treated with cisplatin (33 μM) (Cisp) were used as positive controls for antibody specificity. (e) Bar graph represents mean values obtained by densitometry evaluation of p53 positive bands of three independent experiments ± SD. (f) WB to evaluate NIX expression in UVB‐irradiated Scr and p53 KD HDF. (g) Bar graph represents mean values obtained by densitometry evaluation of NIX positive bands of three independent experiments ± SD. (h) Fibroblasts expressing mRFP (red) irradiated for 2 days (UVB D2) and the corresponding control (Ctrl D2) were fixed and processed for indirect immunofluorescence. NIX was detected with appropriate antibodies and is shown in green. Overlaying of the two channels was obtained by the microscope software and co‐localization is characterized by yellow color (white arrows). Images are representative of three independent experiments. Scale bar: 20 μm. Intensity of NIX nuclear fluorescence (i) and colocalization of NIX (green) and mitochondria (red) (j) in at least 50 cells per group were evaluated by ImageJ software. Graphs represent mean values of three independent experiments ± SD. (k) WB to evaluate NIX expression in control and UVB‐irradiated HDF in the absence and presence of Bafilomycin A. (l) Bar graph represents mean values obtained by densitometry evaluation of NIX positive bands of three independent experiments ± SD. For all experiments *p ≤ 0.05; **p ≤ 0.01; ***p ≤ 0.001; n.s., non‐significant.

To clarify the underlying mechanisms involved in mitophagy activation upon UVB irradiation of HDF, we evaluated the expression of NIX, BNIP3, FUNDC1 and PINK1 in cells submitted to UVB and their respective controls. Whereas UVB irradiation did not significantly affect BNIP3 and FUNDC1 mRNA levels, expression of PINK1 mRNA increased subtly on D4 of the experiment (Figure S2A). Protein levels of BNIP3 were elevated on D2 and decreased on D4 whereas PINK1 protein was consistently downregulated by UVB in comparison with non‐irradiated controls (Figure S2B,C). Expression of FUNDC1 protein was undetectable in Ctrl and UVB‐irradiated HDF. In contrast, expression of the mitophagy receptor NIX was consistently upregulated in response to UVB both at mRNA and protein levels (Figure 3a–c).

As expected, p53 protein expression was increased in response to UVB treatment. Accumulation and stabilization of p53 are considered one of the main responses to DNA damage in the process of UVB‐induced senescence (Anand et al., 2020). Furthermore, dynamics of p53 resembled the one observed for NIX accumulation (Figure 3d,e), supporting the idea that NIX expression is activated by p53 in UVB‐irradiated cells, in line with the fact that the NIX promoter can be activated by p53 in U2OS cells (Fei et al., 2004).

To investigate the regulation of NIX by p53, we generated p53 KD HDF by lentiviral transduction. Efficiency of p53 KD was confirmed by q‐RT‐PCR and WB and p53 mRNA and protein levels were decreased by 80% and 75%, respectively (Figure S2D–F). The expression of NIX protein remained unchanged in p53 KD HDF when compared to Scr HDF on UVB D2. However, NIX protein levels drastically declined in p53 KD HDF in comparison to Scr HDF on UVB D4 (Figure 3f,g) confirming that p53 plays a pivotal role in regulating NIX expression.

To address a potential role for NIX in UVB‐induced mitophagy, we investigated its subcellular localization in control and UVB‐irradiated cells. As observed by confocal microscopy in non‐irradiated cells NIX was mainly present in the nucleus (Figure 3h,i), in line with previous findings by others, reporting NIX localization in the nucleus and endoplasmic reticulum (Chen et al., 2013; Kitamura et al., 2011; Ohi et al., 1999). However, upon 2 days of irradiation, NIX was found to be predominantly colocalized with mitochondria (Figure 3h,j, and Figure S2G). Molecular mechanisms to explain the subcellular re‐localization of NIX remain elusive.

Finally, to assess NIX‐dependent mitophagic flux, we employed the lysosome ATPase inhibitor Bafilomycin A to inhibit the degradation of mitophagosomes by the lysosomes. Notably, upon Bafilomycin A treatment, we observed an accumulation of NIX protein across all experimental groups (Figure 3k,l). Altogether these results suggest that NIX participates in priming damaged mitochondria for clearance by mitophagy in response to UVB irradiation.

### 2.4 Depletion of NIX changes the fate of UVB‐irradiated HDF from senescence to cell death

**FIGURE 4:**
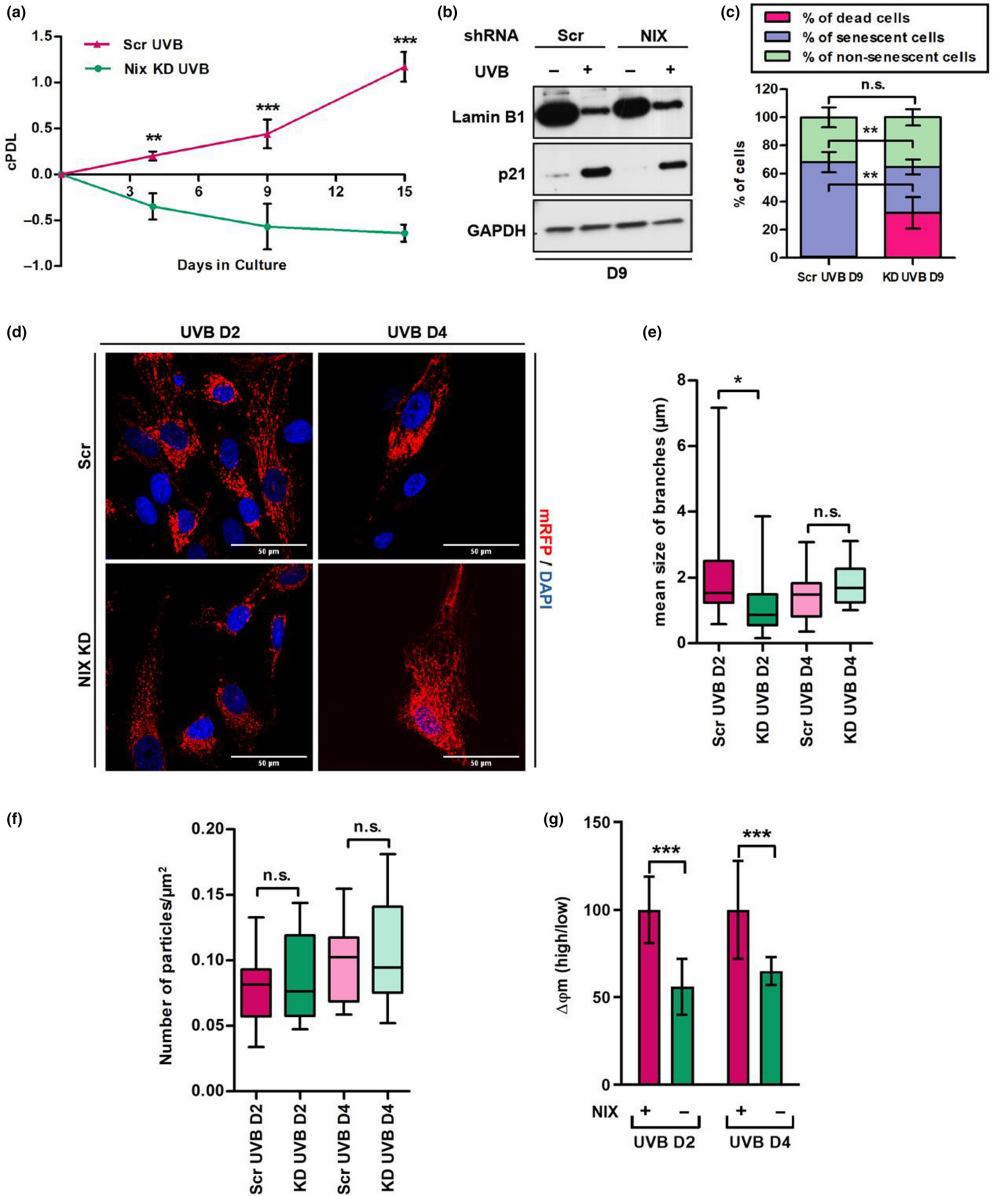
NIX KD leads to sustained mitochondrial damage and changes the fate of UVB‐irradiated fibroblasts from senescence to cell death. Fibroblasts were transduced with lentiviral vectors carrying NIX or scrambled shRNAs and grown under selection. Resulting NIX KD and Scr cells were irradiated twice a day for 4 days and monitored for cell growth (a) expression of senescence‐related proteins (b) and activity of SA‐β‐Gal (c). (a) Cumulative population doublings of the given populations represent mean values of three independent experiments ± SD. (b) WB to evaluate the expression of Lamin B1 and p21 in UVB‐irradiated Scr and NIX‐depleted HDF. GAPDH was used as loading control. Image is representative of three independent experiments. (c) SA‐β‐gal cytochemistry for detection of senescent cells. Blue positive cells were counted, and the percentage of positive cells was calculated dividing the number of positive cells by the total number of cells in a given sample. Number of dead cells was counted by Casy counter. Bars represent mean values of three independent experiments ± SD. For each group at least 400 cells were counted. (d) HDF expressing mRFP were transduced with lentiviral vectors carrying NIX or scrambled shRNAs and grown under selection. The resulting cells were submitted to UVB treatment as described and processed for confocal microscopy. Nuclei were stained with DAPI. Representative images of three independent experiments comparing Scr and NIX KD cells on D2 and D4 of the experiment are shown. (e, f) Analysis of size of mitochondrial branches (e) and number of particles (f) was performed in ImageJ software. Boxes represent minimum, maximum and median length ± SD of mitochondrial branches/particles observed after 2 or 4 days of irradiation. (g) Mitochondrial membrane potential of Scr and NIX KD HDF was accessed by FACS with the use of JC‐1 following 2 and 4 days of UVB. Bars represent the ratio obtained by dividing the percentage of mitochondria with high membrane potential by the percentage of mitochondria with low membrane potential of a given population. Graphic is displayed as mean values of three independent biological samples ± SD. Controls were normalized to 100% for better interpretation of the results. For all experiments *p ≤ 0.05; **p ≤ 0.01; ***p ≤ 0.001; n.s., non‐significant.

To further investigate the role of NIX as a mitophagy receptor during UVB‐induced senescence we employed HDF transduced with lentiviral vectors carrying shRNAs directed to inhibit NIX gene expression (NIX KD) or with scrambled vectors as control (Scr) (Figure S3A,B). NIX KD and Scr cells were irradiated twice a day for 4 days, as described, and monitored for cell proliferation and cellular senescence. UVB‐irradiated Scr cells underwent less than two population doublings during the 15 days of experiment with no apparent cell death. In contrast, UVB treatment of NIX‐depleted HDFs resulted in a significant decrease of cell numbers and negative cPDL in the same time frame (Figure 4a), indicating cell death. Senescence status was further assessed by determining the regulation of senescence‐related proteins Lamin B1 and p21 (Figure 4b and Figure S3C,D) and by determining the activity of senescence‐associated ß‐galactosidase (SA‐β‐Gal) (Figure 4c and Figure S3E). UVB irradiation reduced the expression of Lamin B1 and increased the levels of p21 protein both in NIX KD and Scr HDF. Intriguingly, baseline levels of Lamin B1 protein were already lower in non‐irradiated NIX KD HDF suggesting a predisposition toward premature senescence (Figure 4b and Figure S3C,D). When analyzing the activity of SA‐β‐Gal and considering the number of viable cells remaining after UVB treatment we observed that in the Scr group, more than 99% of the cells survived the UVB treatment. Of these cells, 67% were positively stained for SA‐β‐Gal while 31% remained as viable proliferating cells. In contrast, in UVB‐irradiated NIX KD HDF more than 32% of the population consisted of non‐viable/dead cells, while 32% were positively stained for SA‐β‐Gal and around 35% remained as viable non‐stained cells (Figure 4c and Figure S3E). This shifting of the cell fate from senescence to cell death by NIX knock‐down suggested that NIX—probably due to its function in inducing mitophagy—is an important player in the switch between senescence and cell death of HDF in conditions of stress, such as UVB irradiation.

Accumulation of damaged mitochondria can trigger the release of proteins that participate in cell death pathways (Gao et al., 2020; Kubli & Gustafsson, 2012). Having observed that a considerable percentage of NIX KD cell population could not survive UVB treatment, we next evaluated mitochondrial network morphology in UVB‐irradiated Scr and NIX KD cells transduced with a mRFP reporter. As observed by confocal microscopy UVB‐irradiated Scr cells presented more interconnected mitochondria in comparison to NIX KD cells, especially on D2 of the experiment (Figure 4d–f). Accordingly, UVB‐irradiated NIX KD cells presented a higher, although not significant, number of fragmented mitochondria in the cytoplasm (Figure 4f). Additionally, by assessing mitochondrial membrane potential by JC‐1 staining, we observed that mitochondria of UVB‐irradiated NIX KD cells had a lower membrane potential in comparison to Scr cells (Figure 4g), confirming the persistence of mitochondrial damage in the absence of NIX and reinforcing its role in mitochondrial quality control upon UVB irradiation.

### 2.5 NIX KD delays autophagy and impairs mitophagy in response to UVB

Given that NIX is a selective autophagy receptor and *knockdown* of NIX led to persistence of mitochondrial damage in UVB‐irradiated fibroblasts, we assumed that mitophagy would be impaired in NIX KD cells. To experimentally test this hypothesis, we employed NIX KD and Scr cells that were transduced with lentiviral vectors for the overexpression of GFP‐LC3 generating GFP‐LC3/NIX KD and GFP‐LC3/Scr cells. Transduced cells were irradiated, labeled with Complex V antibody by IF and observed by confocal microscopy analysis, as described before (Figure 1).

**FIGURE 5:**
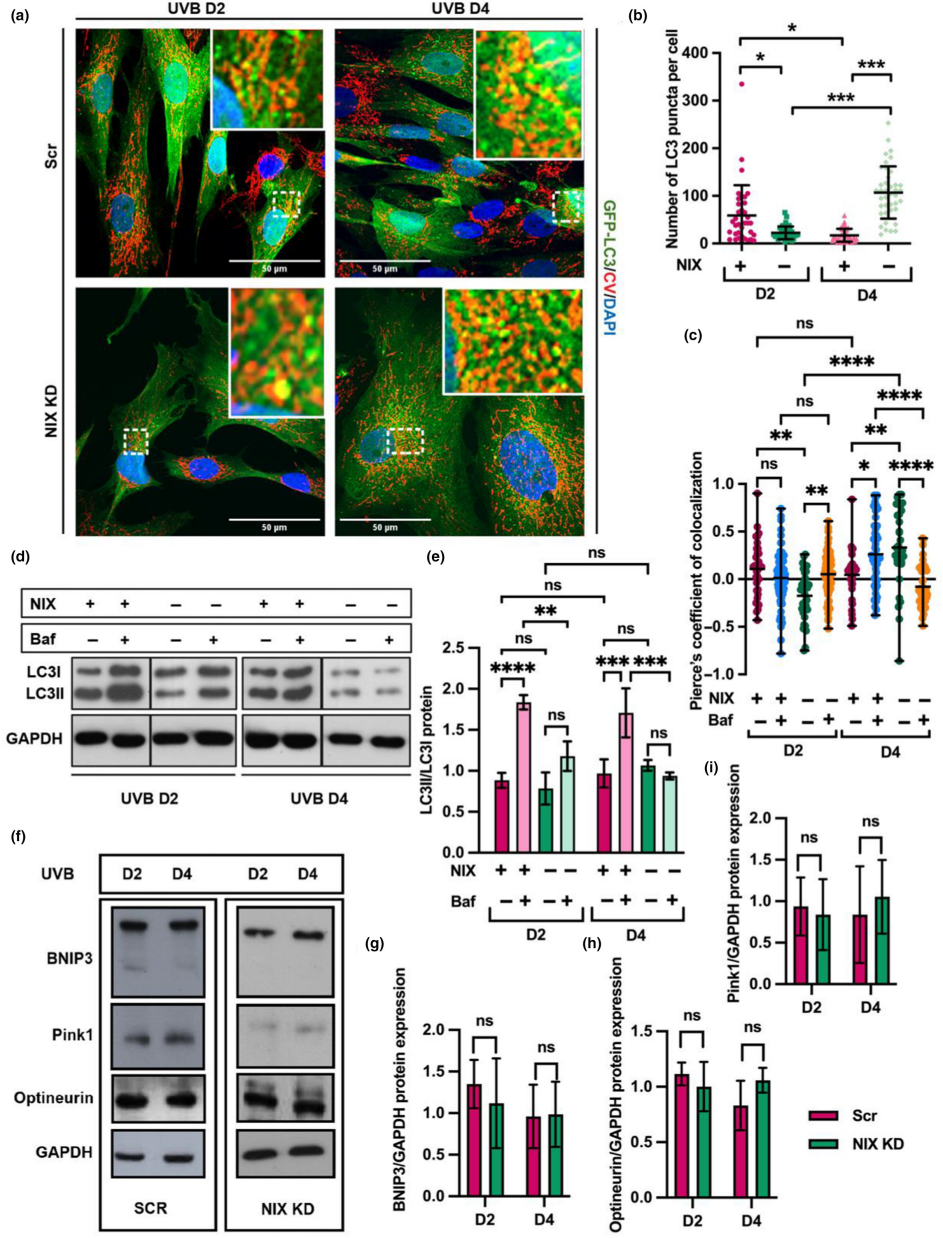
Mitophagy is impaired in NIX KD HDF. (a) HDFs expressing GFP‐LC3 were transduced with lentiviral vectors carrying NIX or scrambled shRNAs and grown under selection. The resulting cells were submitted to UVB treatment as described fixed and processed for indirect immunofluorescence and analyzed by confocal microscopy. Mitochondrial Complex V (CV) was detected with appropriate antibodies and is shown in red, nuclei were stained with DAPI. Representative images of three independent experiments comparing Scr and NIX KD cells on D2 and D4 of the experiment are shown. Enlarged detailed images of mitochondria colocalized with LC3 positive puncta are shown in red squares. Single channels for each picture are displayed in Figure S4A. (b) Mean number of autophagosomes per cell was quantified by ImageJ software and is represented as mean value ± SD. (c) Analysis of colocalization between mitochondria (red) and autophagosomes (green) in NIX KD and Scr cells submitted to 2 or 4 days of UVB, in the presence or absence of Bafilomycin A. Values of the Pearson coefficient above 0 represent positive colocalization and values under 0 represent negative colocalization. (d) WB to detect LC3I and LC3II in UVB‐irradiated Scr and NIX KD HDF in the absence and presence of Bafilomycin A. GAPDH was used as loading control. Images are representative of three independent experiments. (e) Densitometry of bands obtained by WB was performed in Image J software. Results are displayed as values obtained for LC3II divided by the values obtained for LC3I following normalization to GAPDH. (f) WB to detect BNIP3, Pink1 and Optneurin in UVB‐irradiated Scr and NIX KD HDF. GAPDH was used as loading control. Images are representative of three independent experiments. (g–i) Densitometry of bands obtained by WB for BNIP3 (g) Pink1 (h) and Optneurin was performed in Image J software. Graphs represent mean values of 3 independent experiments ± SD normalized to GAPDH. For all graphs: *p ≤ 0.05; **p ≤ 0.01; ***p ≤ 0.001; ****p ≤ 0.0001; n.s., non‐significant.

Scr cells showed a transient increase of LC3‐puncta in response to UVB, with the highest number of spots being observed at the end of the second day (D2) of treatment and declining to the end of the irradiations (D4). In contrast, in NIX KD cells a significant increase in the number of LC3 puncta was only observed on the fourth day of the experiment (D4), 2 days later than the peak detected for Scr cells (Figure 5a,b and Figure S4A,B). Although autophagy activation was delayed, UVB‐irradiated NIX KD cells on Day 4 exhibited the highest count of autophagosomes per cell compared to all other groups (Figure 5b and Figure S4B). A similar observation was made when we evaluated the colocalization of mitochondria and autophagosomes. In UVB‐irradiated Scr cells mitochondria were preferentially colocalized with autophagosomes on D2. In contrast, in UVB‐irradiated NIX KD cells, increased colocalization of mitochondria and LC3 puncta was observed after the last irradiation only, that is, on D4 of the experiment (Figure 5c).

Considering that accumulation of autophagosomes can reflect increased autophagy as well as impairment of autophagic flux, we blocked the fusion of autophagosomes and lysosomes of UVB‐irradiated Scr and NIX KD cells with Bafilomycin A and analyzed the accumulation of the lipidated form of LC3, LC3 II, a reliable method to monitor autophagic flux by WB (Figure 5d,e). Bafilomycin treatment led to a further increase in the proportion of LC3 II/LC3 I in UVB‐irradiated Scr HDF on D2 and D4. In contrast, in NIX‐depleted cells bafilomycin treatment did not increase the accumulation of LC3 II, suggesting an impairment of autophagy upon depletion of NIX. Additionally, when blocking autophagic flux with Bafilomycin A, we observed no difference in the number of autophagosomes when comparing UVB‐irradiated Scr and NIX KD HDF (Figure S4C). Furthermore, in UVB‐irradiated NIX KD HDF blocking of mitophagic flux did not reflect in increased colocalization of mitochondria and autophagosomes as observed in UVB‐irradiated Scr HDF (Figure 5c).

Based on these results, we wondered whether impairment of NIX‐dependent mitophagy would be compensated by the triggering of an alternative mitophagy pathway. Therefore, we evaluated the expression of the mitophagy receptors and related genes PINK1, BNIP3, and FUNDC1 in UVB‐irradiated Scr and NIX KD cells by q‐RT‐PCR and protein expression of the mitophagy receptors BNIP3, Pink1 and Optineurin by WB (Figure S4D and Figure 5f–i). We found no changes in the expression of these genes upon UVB treatment (Figure S4D). Likewise, depletion of NIX did not induce the expression of BNIP3, Pink1, and Optineurin proteins (Figure 5f–i). Altogether our results suggest that depletion of NIX leads to impairment of macroautophagy and mitophagy in UVB‐irradiated HDF and that these are not compensated by alternative mitophagic pathways.

### 2.6 Release of extracellular vesicles may compensate for impaired NIX‐dependent mitophagy in UVB‐irradiated fibroblasts

**FIGURE 6:**
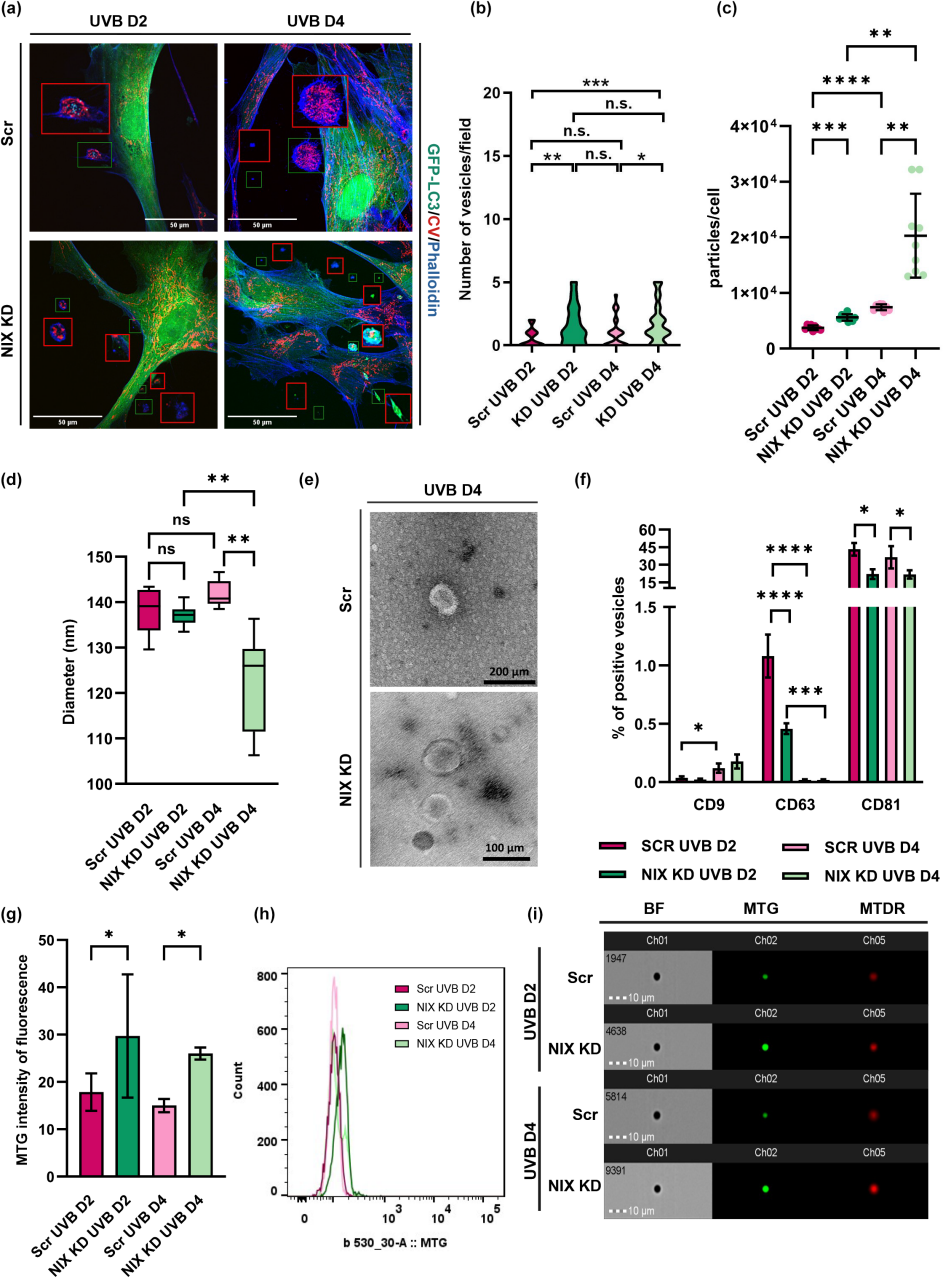
Release of EVs containing mitochondria is increased in response to impairment of mitophagy. HDFs expressing GFP‐LC3 transduced with lentiviral vectors carrying NIX or Scr shRNAs were irradiated for 2 and 4 days, stained with Complex V antibody and phalloidin blue, and observed by confocal microscopy to evaluate the release of vesicle‐like structures. Single channels for each picture are displayed in Figure S5. (a) Images are representative of at least 3 independent experiments. Enhanced detailed images of vesicles are shown in red squares. (b) Violin plot represents the distribution of vesicle‐like structures per microscopic field for each group. (c–g) Evs were isolated by size‐exclusion chromatography and concentration I and size (d) of particles were determined by NTA. (e) TEM of EVs isolated from UVB‐irradiated Scr and NIX KD supernatant. (f) Characterization of EVs tetraspanins CD9, CD63 and CD81 was performed by FACS. (g) Mitochondrial content of EVs was assessed by FACS using the probe Mitotracker Green (MTG). (h) Representative histogram of MTG intensity of fluorescence obtained by MTG staining of UVB‐irradiated Evs. (i) ImageStream analysis of EVs content using mitochondrial probes MTG and Mitotracker Deep Red (MTDR) For all graphs: *p ≤ 0.05; **p ≤ 0.01; ***p ≤ 0.001; ****p ≤ 0.0001; n.s., non‐significant.

Extracellular vesicles (EVs) are released from almost all cell types and are known to participate in processes such as intercellular communication and senescence induction (Terlecki‐Zaniewicz et al., 2018, 2019; Weilner, Keider, et al., 2016; Weilner, Schraml, et al., 2016). Recently, the release of mitochondria‐containing vesicles and free forms of extracellular mitochondria have been suggested as alternative mitochondrial quality control pathways to compensate for perturbations of mitophagy (Choong et al., 2020; Jiao et al., 2021). Hence, we investigated whether UVB would induce the release of EVs and whether the number of such vesicles would be influenced by the absence of NIX. As observed by confocal microscopy, LC3‐positive, Complex V‐positive, and phalloidin‐positive extracellular vesicle‐like structures were present both in preparations from UVB‐irradiated Scr as well as in NIX KD HDF; however, in a significantly higher proportion in NIX KD cells (Figure 6a,b and Figure S5). Therefore, we isolated EVs from conditioned medium by size exclusion chromatography (SEC) and monitored concentration and size of vesicles by nanotracker particle analysis (NTA) (Figure 6c,d). Depletion of NIX significantly increased the release of EVs in UVB‐irradiated HDF, evident in both UVB D2 and UVB D4, with the maximum EV count observed in NIX KD UVB D4 cells (Figure 6c). Remarkably, the size of vesicles was consistently smaller in UVB‐irradiated NIX KD HDF compared to Scr HDF (Figure 6d), a finding corroborated by electron microscopy (Figure 6e). Further characterization of EVs surface markers, including the tetraspanins CD9, CD63, and CD81 by FACS, revealed distinct patterns and confirmed the release of bona fide EVs of which the majority were enriched in CD81 (Figure 6f).

Next, we explored whether EVs released by UVB‐irradiated HDF would carry mitochondria and whether this process would be influenced by the accumulation of damaged mitochondria resulting from NIX inhibition. To address this, EVs were labeled with the mitochondrial probe mitotracker green (MTG), which specifically interacts with thiol groups of mitochondrial proteins, and fluorescence intensity was assessed by FACS. We identified EVs containing mitochondria across all experimental groups. Notably, EVs released by UVB‐irradiated NIX‐depleted HDF were found to contain a greater number of mitochondria when compared to those released by UVB‐irradiated Scr HDF (Figure 6g,h). These observations were further validated by ImageStream analysis of EVs using the fluorescent probes MTG and mitotracker deep red (MTDR) confirming that EVs released by UVB‐irradiated NIX KD HDF are enriched in mitochondria (Figure 6i). Collectively, these findings suggest that the release of EVs containing mitochondria is a potential alternative mechanism of mitochondria quality control, compensating for the inhibition of canonical NIX‐dependent mitophagy.

## 3 DISCUSSION

Photoaging of the skin occurs due to UV irradiation, in particular UVA and UVB. UVB is harmful to biological tissues since it can directly damage organelles and molecules such as mitochondria, nucleic acids, and proteins. Mitochondria, the major sites of intracellular energy metabolism, are directly affected by UVB resulting in DNA damage and generation of ROS (Masaki et al., 2009). UVB rays can cross the epidermis and reach the upper dermis where they interact with cellular chromophores leading to cumulative damage and ultimately to the process of skin aging (Hoffmann et al., 2000; Sanches Silveira & Myaki Pedroso, 2014) However, previous studies relating mitochondria and UVB are primarily focused on the damage caused to keratinocytes and the epidermis (Dhar et al., 2019; Masaki et al., 2009), while very little is known about the effects of UVB on mitochondria in dermal cells. Our study provides new, important insights into the mechanisms of mitochondrial quality control during the process of UVB‐induced senescence of fibroblasts in the context of UVB‐induced photoaging of the skin. We have demonstrated that mitophagy is the main mechanism of mitochondrial quality control activated in HDF upon UVB‐induced damage. Mitophagy in this context was mediated by the receptor NIX and was induced upon mitochondrial damage and fragmentation. Finally, in addition to mitophagy, UVB induced the release of extracellular vesicles enriched in mitochondria. We propose that the shedding of extracellular vesicles containing mitochondria and/or fragments thereof is an alternative mechanism of mitochondrial quality control in NIX KD cells, which compensates, at least in part, the impairment of NIX‐mediated mitophagy upon UVB irradiation. Thus, our findings demonstrate the importance of mitophagy as a critical regulator of cell homeostasis in UVB‐induced senescence and skin photoaging and suggest the existence of a functional interplay between different mitochondrial quality assurance pathways.

Mitochondrial quality control is key to maintaining properly functioning mitochondria, which in turn are necessary for adaptive metabolism and survival in response to cellular stress (Zachari & Ktistakis, 2020). Mitophagy can be activated upon several stimuli and plays an important role in cellular homeostasis by eliminating dysfunctional mitochondria and reducing mitochondrial mass as an adaptive response to stress (Bakula & Scheibye‐Knudsen, 2020; Springer & Macleod, 2016). Acute oxidative stress promotes extensive mitochondrial fission and dysfunction. Accordingly, some reports have suggested that regulated fission of mitochondria can act as a surveillance mechanism to identify poorly functioning organelles and that this process is essential for mitochondrial degradation by mitophagy as it enables the separation of depolarized mitochondria from the mitochondrial network facilitating their engulfment by autophagosomes (Gomes & Scorrano, 2013; Kraus et al., 2021). In UVB‐irradiated HDF, mitophagy was induced in a transient way following increased ROS levels, extensive structural damage of mitochondria visualized by EM, decreased mitochondrial membrane potential, and fragmentation of mitochondrial network. These results are in line with previous findings that demonstrate that UVB leads to mitochondrial fragmentation in human epidermal keratinocytes (Jugé et al., 2016), although in keratinocytes fragmentation of mitochondria following UVB treatment induces bulk autophagy but not mitophagy (Hegedűs et al., 2019), which suggests that different cell types of skin act distinctly to the same type of stress. Consistent with our previous publication, where we showed that the highest number of autophagosomes is reached on D2 of the UVB‐induced senescence model (Cavinato et al., 2016), the peak of mitophagy in response to UVB in HDF was observed on the second day of treatment simultaneously to the appearance of disrupted (“blebbing”) mitochondria. During the development of the senescent phenotype starting from day 4 when UVB irradiation was discontinued, mitophagic activity was continuously declining. Thus, mitophagy acts as an emergency mechanism to prevent the accumulation of damaged mitochondria in the early stages of UVB‐induced senescence of HDFs.

NIX, a ubiquitin‐independent mitophagy receptor is required for the selective removal of mitochondria during the terminal differentiation of reticulocytes (Sandoval et al., 2008). NIX, a member of the BH3 family of proteins, was previously described to participate in two distinct phases of carbonyl cyanide m‐chlorophenyl hydrazone (CCCP)‐induced mitophagy in murine embryonic fibroblasts—induction of autophagy in response to ROS and by promoting Parkin translocation to mitochondria, thus priming them for elimination (Ding et al., 2010). In these experiments, authors concluded that the C‐terminal domain of NIX, including the transmembrane domain, is required for both mitochondrial localization of NIX and its participation in CCCP‐induced RFP‐LC3 punctuation (Ding et al., 2010). Others have suggested that the BH3 domain in the N‐terminal part of NIX is required for autophagy activation, possibly by interfering with Beclin1/Bcl2 complexes (Ding et al., 2010). More recent studies revealed the role of NIX by promoting the formation of a mitochondria‐NIX‐LC3‐autophagosome complex driving mitophagy in a cellular model for spinal cord injury (Nie et al., 2021). The mechanism of autophagy induction through NIX has only been partly resolved and, to the best of our knowledge, activation of NIX‐mediated mitophagy in response to UVB treatment of HDF was not yet reported. We demonstrated here that UVB‐induced mitophagy of HDFs is regulated by NIX not just by increased expression, as described in previous studies (Koentjoro et al., 2017; Lampert et al., 2019), but also by translocation of the protein to the sites of damaged mitochondria. Importantly, we have also demonstrated that NIX expression is induced by p53, confirming previous observations which demonstrated that NIX is controlled by p53 activity (Fei et al., 2004). Blocking of NIX‐dependent mitophagy by NIX KD changed the fate of UVB‐irradiated cells from senescence to cell death suggesting that regulation of mitophagy is crucial for the establishment of the senescence phenotype in UVB‐irradiated fibroblasts. We have previously found that *knockdown* of the autophagy‐related protein Atg7 likewise changes the fate of UVB‐irradiated HDF from senescence to apoptosis (Cavinato et al., 2016). Accordingly, in *C. elegans*. DCT‐1, which is the putative orthologue to the mammalian NIX/BNIP3L and BNIP3, was reported to be a key mediator of mitophagy under conditions of stress and knockdown of this gene decreased the lifespan of long‐lived mutant worms (Palikaras et al., 2015). Finally, it may be of interest that NIX knockdown in mouse auditory cells, used as a model of age‐related hearing loss led to inhibition of mitophagy and subsequent induction of cellular senescence (Kim et al., 2021). It is important to highlight that in our study, we specifically employed NIX knockdown rather than knockout. This approach enables us to evaluate NIX‐mediated mitophagy in most cells—those with NIX levels below a defined threshold, making them incapable of NIX‐dependent mitophagy. Unlike knockout, knockdown does not entirely abolish NIX activity in all cells, allowing us to capture a nuanced perspective that reflects the varied responses within the cell population.

Whereas NIX plays an important role in the elimination of keratinocytes at late stages of differentiation (Simpson et al., 2021), it was shown that UVB exposure of human keratinocytes stimulates the expression of BNIP3, a protein homologous to NIX, resulting in the induction of autophagy, which is indispensable for the protection of keratinocytes from apoptosis in this context (Moriyama et al., 2017). Along these lines, we propose, that macroautophagy and mitophagy are the potential limiting factors that determine the inability of HDFs to cope with the UVB stress.

In WT UVB‐irradiated HDF, activation of macroautophagy, as well as NIX‐dependent mitophagy, followed increased ROS levels, already on D2 of the experiment. Yet, depletion of NIX delayed and impaired autophagy induction in response to UVB. Thus, in this system, the activation of macroautophagy seems to be at least partially dependent on NIX. Furthermore, we did not detect UVB‐induced meaningful changes in the expression of PINK1, FUNDC1, and Optineurin at either the mRNA or protein level. However, we found increased expression of BNIP3 following 2 days of UVB treatment. BNIP3 and NIX are highly related multi‐functional outer membrane proteins, known to prime mitochondria for mitophagy elimination under different conditions (Field & Gordon, 2022). These results suggest that in UVB‐irradiated HDF NIX directly works as a mitophagy receptor by recruiting LC3 to the sites of damaged mitochondria in a process independent of ubiquitin and PARKIN, consistent with previous observations in other cell types (Wei et al., 2015). Additionally, given their similarity, it remains plausible that BNIP3 may serve as a secondary player in the induction of mitophagy following UVB irradiation of fibroblasts, though the specific mechanisms await further exploration.

Mitophagy is an important, but not the unique mean to eliminate mitochondrial damage. Under certain circumstances, regulatory crosstalk between distinct mitophagy pathways seems to compensate for the failure of one or more processes that would compromise mitophagy execution. For instance, in HeLa cells, PINK1/PARKIN are the main players in mitophagy stimulated by Cadmium. However, in the absence of functional PARKIN, NIX‐mediated mitophagy is induced providing an alternative pathway for the elimination of mitochondrial damage in these cells (Naeem et al., 2020). In our experimental model of UVB‐induced senescence of HDF, a large proportion of NIX KD cells died upon UVB treatment due to their inability to perform NIX‐dependent mitophagy. At the same time, a significant number of cells were able to survive the stress, part of which underwent stress‐induced premature senescence while others remained as cycling cells. In contrast to the findings reported with Cadmium‐treated HeLa cells, UVB‐irradiated NIX KD cells did not compensate for the absence of NIX‐dependent mitophagy with the induction of other mitophagy receptors. This finding reinforces the essential role of this protein in the maintenance of HDFs homeostasis under conditions of stress. Instead, these cells seemed to rely, at least in part on the emission of extracellular vesicles, most of them containing mitochondria, as an alternative mechanism of mitochondrial quality control. Recently, Choong and colleagues have shown that perturbation of mitophagy pathways stimulates extracellular mitochondrial release, while overexpression of mitophagy‐related proteins and receptors such as PARKIN, BNIP3 and NIX suppresses the expulsion of mitochondria (Choong et al., 2020). In our experiments, shedding of EVs was stimulated by UVB treatment and further enhanced in NIX KD cells. Of note, ultrastructural analysis revealed that UVB treatment caused blistering of mitochondrial membrane of wild‐type cells. Blebbing of the mitochondrial membrane was previously reported by others as an early key event in the generation of mitochondria‐derived vesicles in both HeLa cells and mouse embryonic fibroblasts (Soubannier, McLelland, et al., 2012; Sugiura et al., 2014). These findings suggest that mitochondrial blebbing, an aspect of mitochondrial morphology not previously documented in irradiated fibroblasts, may indicate the generation of EVs containing mitochondria. Yet, the necessary conditions for the formation of these vesicles, their composition under different circumstances and their relevance for the processes of cellular communication and senescence have still to be investigated.

In conclusion, we have demonstrated that UVB induces mitochondrial damage in HDFs and this leads to mitophagy activation. Mitophagy, in this context, is regulated by NIX and is essential for the establishment of senescence upon UVB damage. In the absence of NIX‐dependent mitophagy, the release of extracellular vesicles may serve as an alternative mechanism of quality control to ensure the survival of UVB‐irradiated HDF. Altogether our findings demonstrate that mitochondrial quality control mechanisms are essential for the maintenance of skin homeostasis and could be potential targets for intervention to counteract mechanisms of skin aging and disease.

## 4 EXPERIMENTAL PROCEDURES

### 4.1 Chemicals

All chemicals were purchased from Sigma (Steinheim, Germany) unless stated otherwise.

### 4.2 Cell culture and UVB treatment

Human foreskin fibroblasts (HFFs) purchased from ATCC (Manassas, VA) were cultivated in DMEM as described (Hutter et al., 2004). Cumulative population doublings (cPDL) were calculated using the formula cPDL = (log(A) − log(B))/0.301 where A: number of cells at the end of a given passage; B: number of cells seeded at the beginning of the given passage. HEK 293FT cells (Life Technologies #R700‐07) were cultivated in DMEM supplemented with 10% FBS, 0.1 mM MEM Non‐Essential Amino Acids, 6 mM L‐glutamine, 1 mM MEM Sodium Pyruvate, 4.5 g/L Glucose, 1% Pen/Strep, and passaged at least three times prior to lentivirus production. UVB treatment of HDFs was performed as described (Greussing et al., 2013) with 0.05 J/cm^2^ either using the output of a Philips TL20W/01 lamp (Philips, the Netherlands) or of a BIO‐SUN irradiation system (Vilber, Germany). [Correction added on 15 June 2024, after first online publication: The description of the UVB irradiation dose 0.05 J/m² has been corrected as 0.05 J/cm² in this version.] At the end of each irradiation, HBSS was replaced by DMEM. Control cells were washed in HBSS and kept in the irradiation chamber, at the same time as the treated cells, in a position in which they were not affected by UVB.

### 4.3 Cytochemistry for senescence‐associated β‐galactosidase

Appearance of senescent cells was monitored by senescence‐associated β‐galactosidase (SA‐*β‐*gal) activity staining using a standard protocol (Greussing et al., 2013). Blue staining, indicative of SA‐*β‐*gal activity, was documented by phase‐contrast microscopy (Nikon Eclipse TE300 microscope connected to a Nikon Digital Sight DS‐U2 camera – Nikon, Amsterdam, The Netherlands). For every sample at least 20 random pictures were taken. The percentage of positive cells was calculated dividing the number of blue positive cells by the total number of cells contained in a field. At least 400 cells were counted by experiment.

### 4.4 Analysis of oxidative stress and mitochondrial membrane potential by flow cytometry

Mitochondrial ROS and mitochondrial membrane potential were measured by flow cytometry with the use of the fluorescent probes MitoTracker Red (CM‐H_2_XRos; Molecular Probes, Vienna, Austria) and JC‐1 (Fisher Scientific, Vienna, Austria), respectively as described (Cavinato et al., 2016). Briefly, cells were trypsinized, counted, and resuspended in medium containing either 0.5 μg/mL JC‐1 or 100 nM CM‐H_2_XRos for 30 min at 37°C. After incubation, cells were washed and resuspended in phosphate‐buffered saline. Fluorescence was measured using the fluorescence‐activated cell sorting (FACS) Canto II flow cytometer (BD Biosciences, Franklin Lakes, NJ). As a positive control for JC‐1, 5 μM of the mitochondrial uncoupler FCCP was used along with the staining. The positive control for CM‐H_2_XRos consisted of cells incubated with 0.5 μM of the mitochondrial complex I inhibitor rotenone along with the staining.

### 4.5 Protein isolation and western blotting

Whole‐cell lysates were separated by SDS‐PAGE gel electrophoresis and transferred to PVDF membranes using standard protocol (Greussing et al., 2013). The primary antibodies used were rabbit monoclonal anti‐BNIP3L/NIX (Cell Signaling, Danvers, MA, #12396S), mouse monoclonal anti‐p53 (Santa Cruz Biotechnology, Dallas, TE, #sc‐126 [Do‐1]), rabbit polyclonal anti‐Lamin B1 (Abcam, Cambridge, UK #ab16048), rabbit monoclonal anti‐p21 (Cell Signaling, Danvers, MA, #2947), rabbit polyclonal anti LC3I/LC3II (Cell Signaling, Danvers, MA, #4108), Rabbit monoclonal anti‐BNIP3 (Cell Signaling, Danvers, MA, #44060), rabbit monoclonal anti‐Pink1 (Cell Signaling, Danvers, MA, #6946), rabbit monoclonal anti‐Optineurin (Cell Signaling, Danvers, MA, #58981), rabbit polyclonal anti‐pRB (BD, Germany, #554136), rabbit polyclonal anti‐GAPDH (Santa Cruz Biotechnology, Dallas, TE, #sc‐25778). Secondary polyclonal HRP‐conjugated antibodies from Dako were used in all cases.

### 4.6 RNA isolation and quantitative real‐time PCR

Total RNA was extracted from cells using the RNeasy® Mini Kit (Qiagen, #74106) according to the manufacturer's protocol. RNA concentration was quantified by photometric measurement at 260 nm. cDNA synthesis was performed using the RevertAid First Strand cDNA Synthesis Kit® (Thermo Scientific #K1622) according to the manufacturer's protocol. Amplification of different genes was performed using the following primers NIX FW: 5′‐GCA AGC AAG AGA AAG CTG GT‐3′ RE: 5′‐CGG TCT CTG GTT GAA TCT CC‐3′; BNIP3: FW: 5′‐GCCATCGGATTGGGGATCTAT‐3′ RE: 5′‐ GCCACCCCAGGATCTAACA‐3′; FUNDC1: FW: 5′‐CCCCTCCCCAAGACTATGAAAG‐3′ RE: 5′‐CGAACTGTGGCCAAACACTC‐3′ and PINK1: FW: 5′‐ TGCAGTGCTGCTGTGTATGA‐3′ RE: 5′‐ GAACCTGCCGAGATGTTCCA‐3′. GAPDH was used as housekeeper and amplified with the primers FW: 5′‐ GAG TCA ACG GAT TTG GTC GT‐3′ and RE: 5′ GAT CTC GCT CCT GGA AGA TG‐3′. Quantitative real‐time PCR was performed in LightCycler® 480 (Roche Applied Science) using SYBR Green as dye.

### 4.7 Manipulation of gene expression in HDF by lentiviral vectors

GFP‐LC3 lentiviral vectors were prepared by insertion of the plasmid containing the human gene MAP1LC3A/LC3 (Microtubule‐associated proteins 1A/1B light chain 3A) fused at its 5′ end to the green fluorescent protein (GFP) (Addgene, Cambridge, MA plasmid #11546) into the pLKO.1 puro (Addgene, Cambridge, MA plasmid #8453) empty backbone. mRFP lentiviral vectors were prepared by insertion of the plasmid containing the leader sequence E1‐alpha of human mitochondrial pyruvate dehydrogenase fused to the red fluorescent protein (RFP) into the pLKO.1 puro (Addgene, Cambridge, MA plasmid #8453) empty backbone as described (Greussing et al., 2012; Pircher et al., 2011).

For stable knockdown, lentiviral vectors carrying NIX‐targeting shRNA sequence were produced as described (Muck et al., 2008; Pircher et al., 2011). HFFs were seeded at 80% confluence in 6 well plates and incubated overnight. On the next day, medium was replaced by DMEM containing 5 MOI of virus plus 8 μg/mL Polybrene. Lentiviruses containing scrambled vectors (Scr) were used as controls. 24 h after infection, the media was exchanged. Selection with puromycin (500 ng/mL) started on day three after infection. For stable overexpression U2OS cells were infected with 6MOI of viral particles and selected with 10 μg/mL blasticidin for 12 days as described (Greussing et al., 2013). For stable p53 knockdown HDF were transduced with p53 shRNA (h) lentiviral particles (Santa Cruz Biotechnology, #sc‐29435‐V).

### 4.8 Electron microscopy

Fibroblasts were processed for electron microscopy as described in (Frank et al., 2002). Briefly, cells were washed, trypsinized and fixed in suspension with Karnovsky's formaldehyde‐glutaraldehyde fixative. All specimens were post‐fixed in aqueous 3% osmium tetroxide and contrasted “en bloc” with 0.5% veronal‐buffered uranyl acetate. Samples were then dehydrated and embedded in Epon 812 resin. Ultrathin sections were mounted on nickel grids, contrasted with lead citrate and were examined in the transmission electron microscope (Phillips EM 400; Fei Company Electron Optics, Eindhoven, the Netherlands) at an operating voltage of 80 kV.

### 4.9 Immunostaining of cells grown on coverslips

After the indicated treatments, cells were fixed with 4% paraformaldehyde and processed for immunofluorescence using standard protocols (Pircher et al., 2011). The following primary antibodies were used: monoclonal mouse anti‐ATP Synthase beta (Thermo Fisher, Vienna Austria, A21351), polyclonal rabbit anti‐BNIP3L/NIX (Abcam, Cambridge, UK, ab8399). Secondary antibodies conjugated Alexa Fluor (Thermo Fisher Scientific, Waltham, MA) were used for these experiments. For observation of cytoskeleton proteins cells were stained with Alexa Fluor 350 Phalloidin (Thermo Fisher Scientific, Vienna Austria, A22281). Stained slides were mounted in fluorescence mounting medium (Dako Cytomation, Copenhagen, Denmark). Images were acquired on Cell Voyager CV1000 (Visitron Systems, Puchheim, Germany) and analyzed using the ImageJ software.

For gated STED (gSTED) super‐resolution confocal microscopy, coverslips were prepared and incubated with primary antibodies as described above. Abberior STAR ORANGE goat anti‐rabbit IgG and Abberior STAR RED goat anti‐mouse IgG (Abberior GmbH, Goettlingen, Germany, 2‐0032‐051‐9) were used as secondary antibodies. Counterstaining with DAPI was omitted and slides were mounted with Abberior Mount Liquid Antifade and dried overnight. Images were acquired on Olympus IX‐83 equipped with a Nanoscope and confocal microscope (STEDYCON) with tunable pulsed STED laser (775 nm), 405 nm (cw), 488 nm, 561 nm and 640 nm pulsed excitation laser lines, 100×/1.4 Oil objective (Abberior GmbH, Goettlingen, Germany). Deconvolution of gSTED images was performed with Huygens Professional (Scientific Volume Imaging, Hilversum, the Netherlands).

For live‐cell imaging GFP‐LC3/mRFP expressing cells were cultivated in Nunc® Lab‐Tek® 8 well plates (ThermoFisher Scientific, Vienna, Austria) and irradiated as described above. Immediately after irradiation media was supplemented with 10 mM Hepes (Thermo Fisher Scientific, Waltham, MA) and the living cells were observed in a spinning disk confocal system (UltraVIEW VoX; Perkin Elmer, Waltham, MA) connected to a Zeiss Axio Observer Z1 microscope (Zeiss, Oberkochen, Germany). Images were acquired with Volocity software (Perkin Elmer; Waltham, MA). For studies of autophagic flux, 50 nM LysoTracker® Blue (LysoTracker® Blue DND‐22, Thermo Fisher Scientific, Waltham, MA) was added to the media and incubated at 37°C for 5 min prior to microscope analysis.

### 4.10 Image analysis

Image analysis was performed with the Fiji/ImageJ software in a blinded manner using non‐visibly discernible microscopy images. Analysis of mitochondrial fragmentation was performed using the particle analysis macro and the MiNa plugin. Analysis of colocalization was performed by creating a Region of Interest (ROI) in a same cell for each channel and analyzing the Coefficient of Colocalization using the plug‐in Coloc2. Analysis of intensity of fluorescence was performed by calculating the mean gray value of a ROI minus the background value of a region of the same picture containing no structure.

### 4.11 Isolation of EVs by size exclusion chromatography (SEC)

UVB‐irradiated Scr and NIX KD HDF were cultivated in DMEM prepared with EV‐depleted FCS for 72 h prior to EV isolation (Borghesan et al., 2019). Conditioned medium of cells was collected and centrifuged at 350 *g* for 15 min at 4°C to pellet cell debris. The supernatant was further centrifuged at 2000 *g* for 20 min at 4°C to remove big particles and apoptotic bodies. The resulting medium was concentrated by ultrafiltration at 2000 *g* for 30 min using Amicon Ultra‐15 filters (100 K, Millipore, Billerica MA). Resulting supernatant was submitted to SEC using qEV columns (qEVoriginal 70 nm, IZON, USA). 6 fractions of 400 μL were collected by gravity elution with filtered PBS and following characterization of fraction enrichment by NTA were pooled for subsequent analysis.

### 4.12 Nanoparticle tracking analysis (NTA)

Concentration and size of particles eluted from SEC were assessed using ZetaView particle tracking analyzer (PMX‐230‐S‐488/640, Particle Metrix, Diessen, Germany) as described (Terlecki‐Zaniewicz et al., 2019). Data of triplicates were analyzed using the ZetaView analysis software and concentration of particles was normalized to cell numbers.

### 4.13 Affinity‐based capture of EVs on beads and surface marker characterization

For affinity‐based capture of EVs, 3.4 × 10^8^ EVs were mixed with 380 μL PBS and 20 μL of Exosome‐Human CD81 Flow Detection Reagent (Invitrogen, USA, #10622D), overnight at 4°C on shaking plate. The following day, CD81‐captured EVs were washed in filtered PBS and incubated with the antibody mix containing anti‐human REAfinity CD63‐FITC, CD9‐APC‐770 and CD81‐PE (Miltenyi Biotech, Germany, #130‐118‐076, #130‐113‐438, and #130‐118‐342, respectively) at 4°C for 1 h on shake plate. Isotype controls were prepared with REA Control Antibody (S), human IgG1, REAfinity antibodies for each fluorophore. Following incubation, EVs were washed and resuspended in filtered PBS and analyzed in FACScan (FORTESSA, Becton Dickinson).

### 4.14 Characterization of EVs content by FACS and ImageStream

EVs were captured using CD81 Flow Detection Reagent (Invitrogen, USA, #10622D) as described above. For FACS analysis, EVs were stained with 10 nM mitotracker green (Invitrogen, USA, #M7514) for 30 min at 37°C, washed and resuspended in PBS, and analyzed in FACScan (FORTESSA, Becton Dickinson). For ImageStream analysis, EVs were simultaneously stained with 10 nM mitotracker green (Invitrogen, USA, #M7514) and 10 nM mitotracker deep red (Invitrogen, USA, #M22426) for 30 min at 37°C, washed and resuspended in PBS, and analyzed in mageStream®X Mark II Imaging Flow Cytometer (Merk Millipore).

### 4.15 Transmission electron microscopy of EVs

For electron microscopy visualization, EVs were processed and imaged as described previously (Oh et al., 2022). Briefly, formvar‐coated copper grids were placed on a 10 μL sample drop for 5 min. After gently removing excess sample with Whatman paper, the grids were quickly washed with distilled water and subsequently floated in a 10‐min drop of 2% glutaraldehyde for fixation. Following a rapid distilled water wash, negative staining was performed using uranyl acetate replacement stain (UAR_EMS Stain, EMS‐Electron Microscopy Sciences). The grids were floated on a drop of UAR twice for 10 s and a third time for 60 s, gently blotting off excess stain after each step. Grids were allowed to air dry before imaging, conducted using a FEI Tecnai G2 transmission electron microscope operating at 160 kV.

### 4.16 Statistics

All experiments were repeated at least three times. The number of repetitions and the way the results are displayed as well as how the statistical analysis was performed is included in the legend of each figure. Differences were compared using Student's *t*‐test. In all graphics: *p < 0.05; **p < 0.01; ***p < 0.001.

## AUTHOR CONTRIBUTIONS

M.C. conceived and carried out the major part of the experiments. I.M., S.W., A.P., R.W., M.Shoss., M.R.B, E.A., B.J., M.H., N.R., performed experiments/analysis. R.K. partially supervised the work. J.H., C.P., G.P., M.Schm., J.G. provided material for experiments and discussed the results. P.J.D. supervised the work and provided funding. M.C. wrote the manuscript with the input of all co‐authors.

## CONFLICT OF INTEREST STATEMENT

The authors declare that there are no competing interests associated with this manuscript.

## Supporting information


Figures S1–S5.

Video S1.

## Data Availability

The data that support the findings of this study are available from the corresponding author upon reasonable request.
