# Supplementary material for: Elimination of damaged mitochondria during UVB‐induced senescence is orchestrated by NIX‐dependent mitophagy
Source: Aging Cell. 2024 May 17;23(8):e14186. doi: 10.1111/acel.14186 (PMC11320349; doi:10.1111/acel.14186)
Supplement: Supplementary file 1 — Figures S1–S5. [file ACEL-23-e14186-s002.docx]

**Supplementary Files**

**
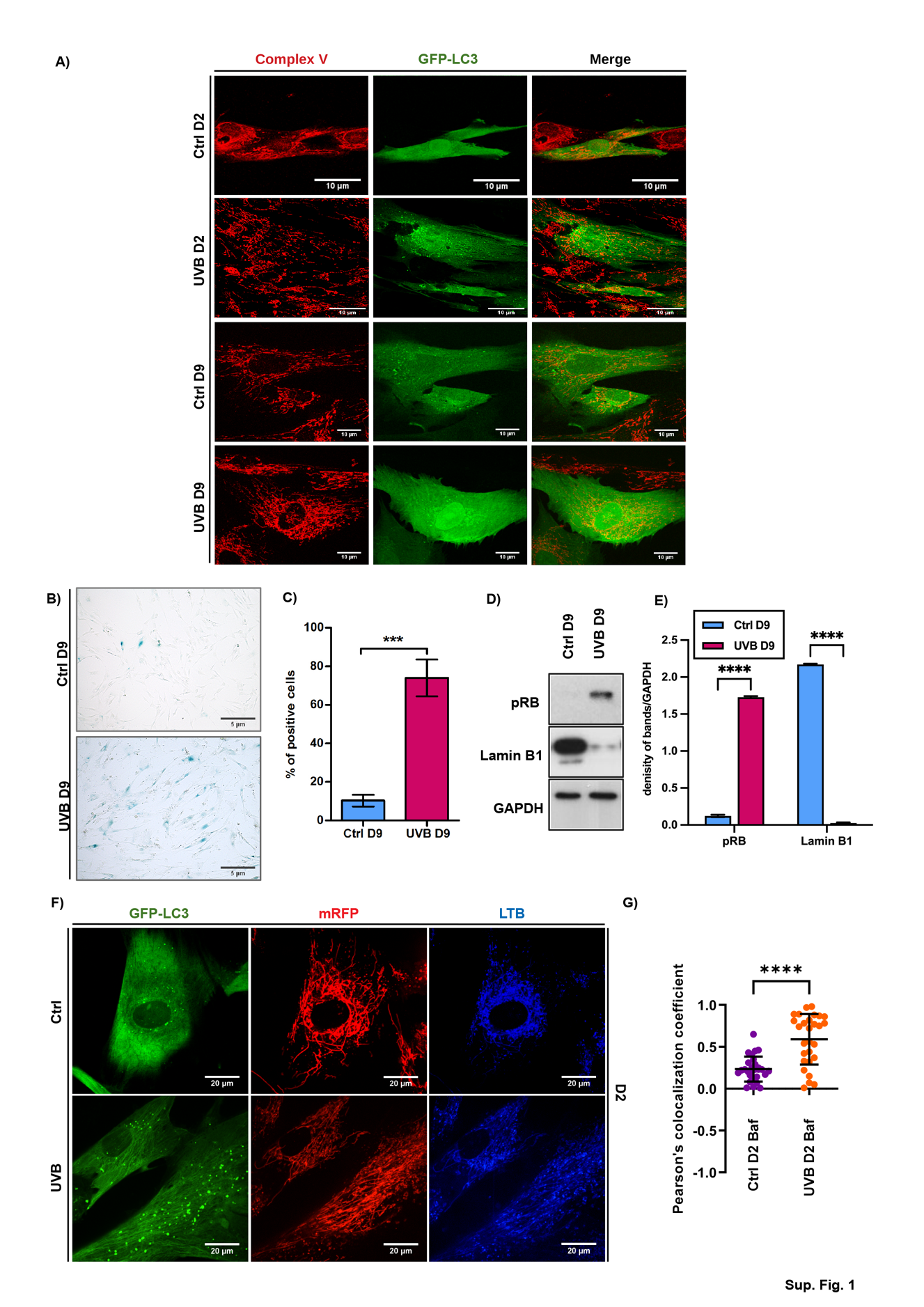
**

**Suplementary Figure 1: Mitochondria damaged by UVB are eliminated by mitophagy.** A) Fibroblasts expressing GFP-LC3 were grown on coverslips and UVB-irradiated. Immediately after the second day of irradiation (D2) or after 4 days of irradiation and 5 days of recovery (D9), cells were fixed and processed for immunofluorescence using anti-complex V antibody–Alexa fluor 594 conjugated (red) and observed under confocal microscope. Scale bar 10 µm. Images from A (UVB D2 and UVB D9) represent single channels of merged picture displayed in figure 2A. B-E) Wild-type fibroblasts were irradiated as described. On D9 after the first irradiation cells were monitored for the activity of SA-β-Gal (B-C) and for the expression of senescence-related proteins by WB (D-E). B) Representative pictures of UVB-irradiated and control cells stained for SA-β-Gal activity. Scale bar 5µm. C) Percentage of SA-β-Gal positive cells was calculated by dividing the number of blue positive cells by the total number of cells in a given area. For each group, at least 400 cells were counted. Bars represent mean values of three independent experiments ± SD. D) Representative WB of Ctrl and UVB-irradiated HDF using appropriate antibodies to detect pRB and LaminB1. GAPDH was used as loading control. E) Densitometry quantification of bands obtained in three independent WBs as shown in (D). F) GFP-LC3/mRFP-expressing fibroblasts exposed to two days of irradiation and the correspondent controls were stained with Lysotracker® Blue and observed by live cell confocal microscopy to investigate the elimination of mitophagosomes by mitophagy. Images represent single channels of merged pictures displayed in Figure 2C. Scale bar 20 µm. G) Analysis of colocalization between mitochondria (red), autophagosomes (green) and lysosomes (blue) in bafilomycin-treated Ctrl and UVB-irradiated HDF was performed in ImageJ software. Values of the Pearson coefficient above 0 represent positive colocalization and values under 0 represent negative colocalization. Results are represented as mean value ± SD of three independent experiments. For all graphs: * p≤0.05 **p≤0.01 ***p≤0.001, ****p≤0.0001 n.s. non-significant.

**
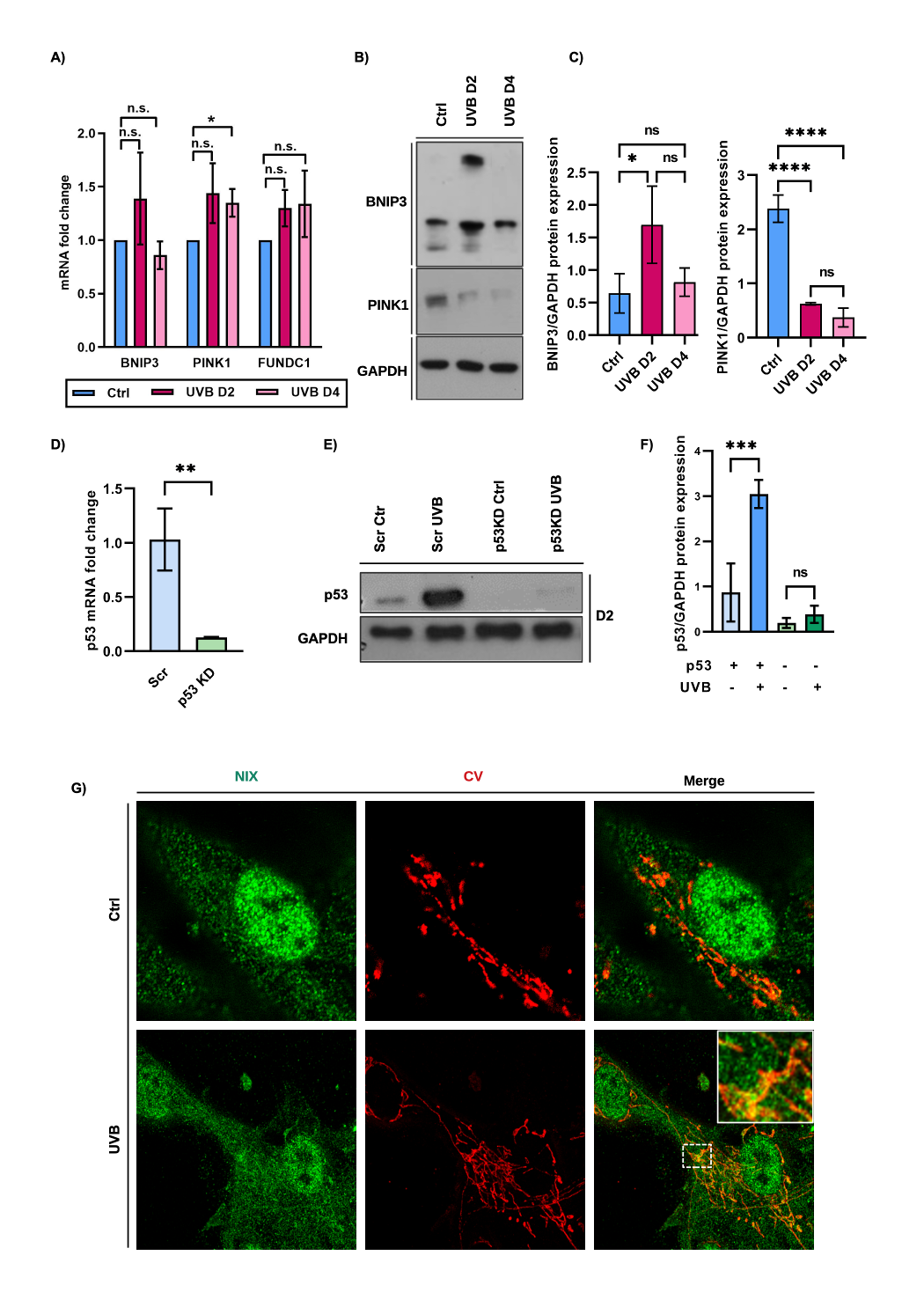
**

**Supplementary Figure 2: Analysis of gene regulation and sub-cellular localization of mitophagy receptors in HDFs submitted to UVB.** A) RNA samples obtained from UVB-irradiated and control HDFs were analyzed by q-RT-PCR to evaluate the effect of irradiation over the regulation of the mitophagy receptors and related genes BNIP3, PINK1 and FUNDC1. (B) Protein lysates obtained from UVB-irradiated and control HDFs were analyzed by WB using antibodies to detect BNIP3 and PINK1. GAPDH was used as loading control. Image is representative of three experiments. C) Densitometry analysis of WB bands for BNIP3 and PINK 1 normalized to GAPDH was performed in ImageJ. D-F) Fibroblasts were transduced with lentiviral vectors carrying p53 or control (scrambled) shRNAs and grown under selection. Expression of p53 was measured by qRT-PCR (D) and WB (E-F). F) Densitometry analysis of WB bands for p53 normalized to GAPDH was performed in ImageJ. G) HDFs submitted to 2 days of UVB treatment, and the corresponding controls were processed for immunofluorescence, labeled with anti-NIX/STAR ORANGE (green) and anti-complex V/STAR RED (red) antibodies, and analyzed by super-resolution microscopy to confirm the change of NIX subcellular localization in response to UVB. Super-resolution microscopy achieves better resolution at greater depths in comparison to confocal microscopy and offers the potential to reveal the subcellular localization and visualization of cellular structures with higher precision. Inset: detail of cytoplasm of irradiated cells showing yellow spots resulting from the colocalization of NIX (green) and mitochondria (red). For all graphs: * p≤0.05 **p≤0.01 ***p≤0.001, ****p≤0.0001, n.s. non-significant.

**
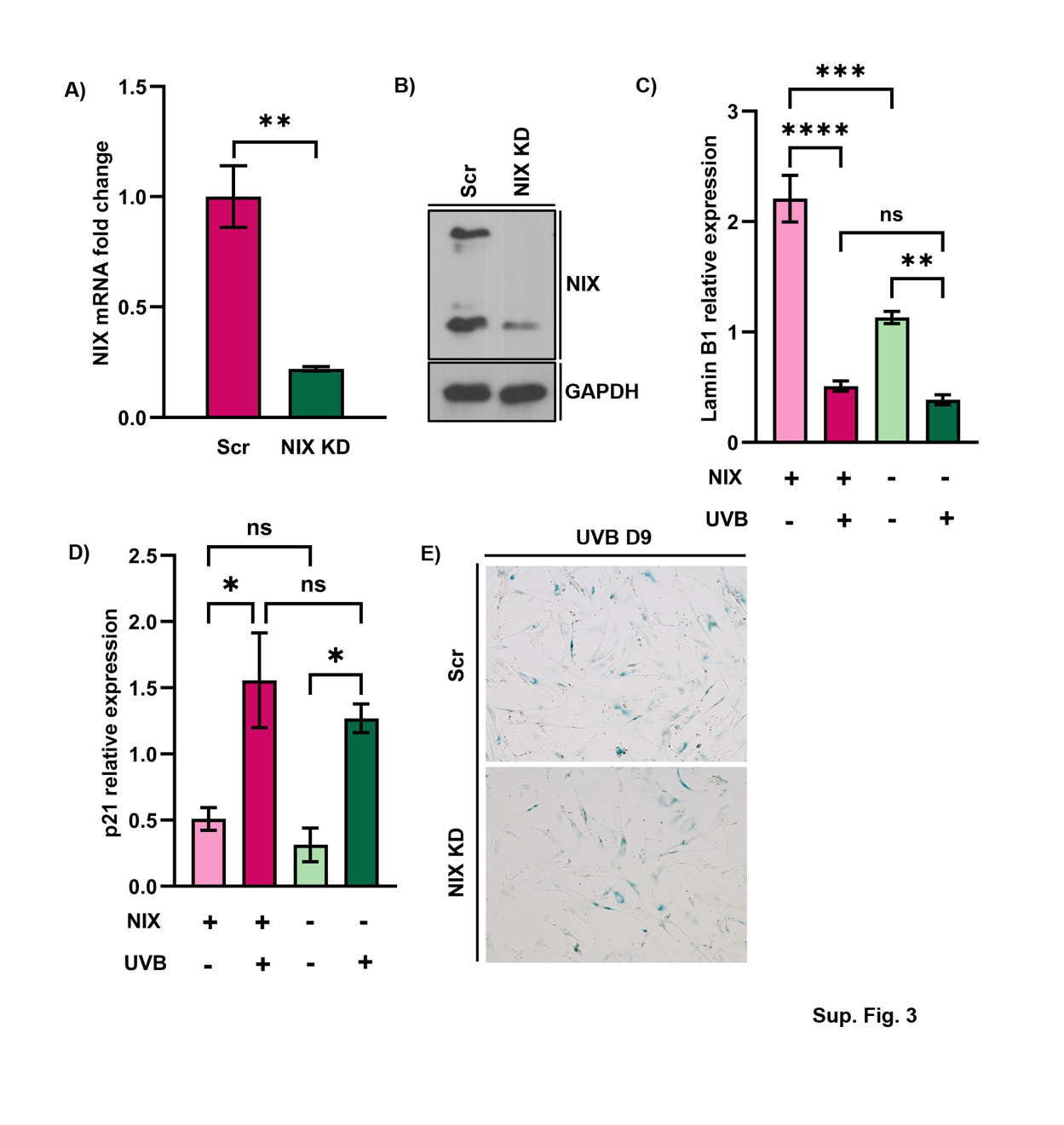
**

**Supplementary Figure 3: NIX KD in HDFs.** A-B) Fibroblasts were transduced with lentiviral vectors carrying NIX or control (scrambled) shRNAs and grown under selection. Expression of NIX was measured by qRT-PCR (A) and WB (B). C-D) Densitometry analysis of WB for Lamin B1 and p21 as shown in Fig. 4B. E) Representative pictures of UVB-irradiated and control Scr and NIX KD cells stained for SA-β-Gal activity. For all graphs: * p≤0.05 **p≤0.01 ***p≤0.001 ****p≤0.0001, n.s. non-significant.

**
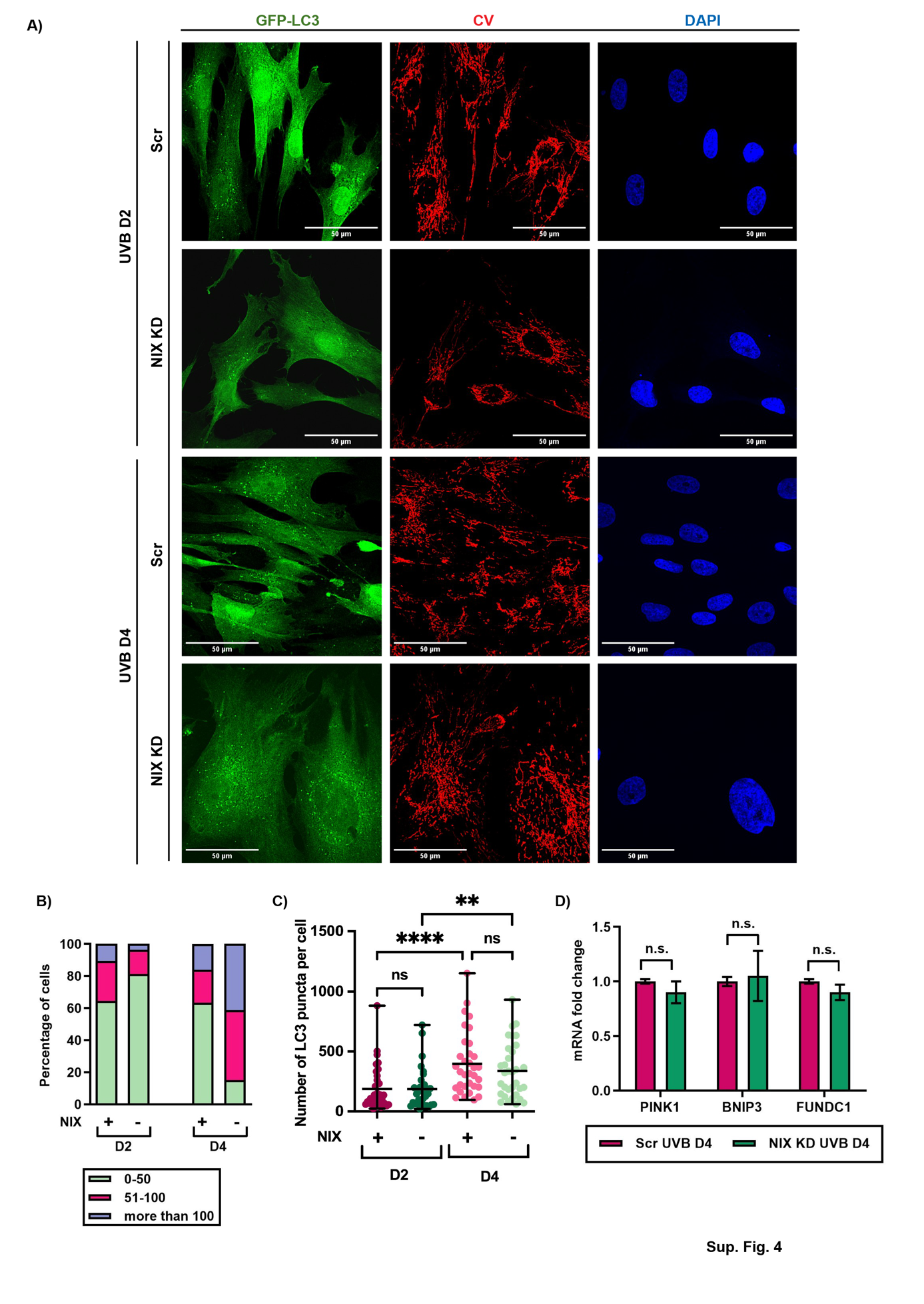
**

**Supplementary Figure 4: NIX KD cells present impairment in autophagy and mitophagy.** GFP-LC3-expressing fibroblasts were transduced with lentiviral vectors carrying NIX or Scr shRNAs. A) Resulting cells were grown in coverslips and UVB-irradiated for 2 or 4 days (UVB D2 and UVB D4, respectively), fixed and labelled with complex V antibody and observed under confocal microscope to evaluate occurrence of autophagy and mitophagy under these conditions. Nuclei were counterstained in blue with DAPI. Images depict separated channels of figure 5A. B) Number of autophagosomes per cell was quantified by ImageJ software. Stacked bar graphs show the distribution of autophagosomes per cells for each given population. C) UVB-irradiated NIX KD and Scr HDF were treated with Bafilomycin A for blocking autophagic flux. Mean number of autophagosomes per cell was quantified by ImageJ software and is represented as mean value ± SD. D) q-RT-PCR analysis of expression of mitophagy-related genes PINK1, BNIP3 and FUNDC1 performed in UVB-irradiated Scr and NIX KD HDF. For all graphs: * p≤0.05 **p≤0.01 ***p≤0.001 ****p≤0.0001, n.s. non-significant.

**
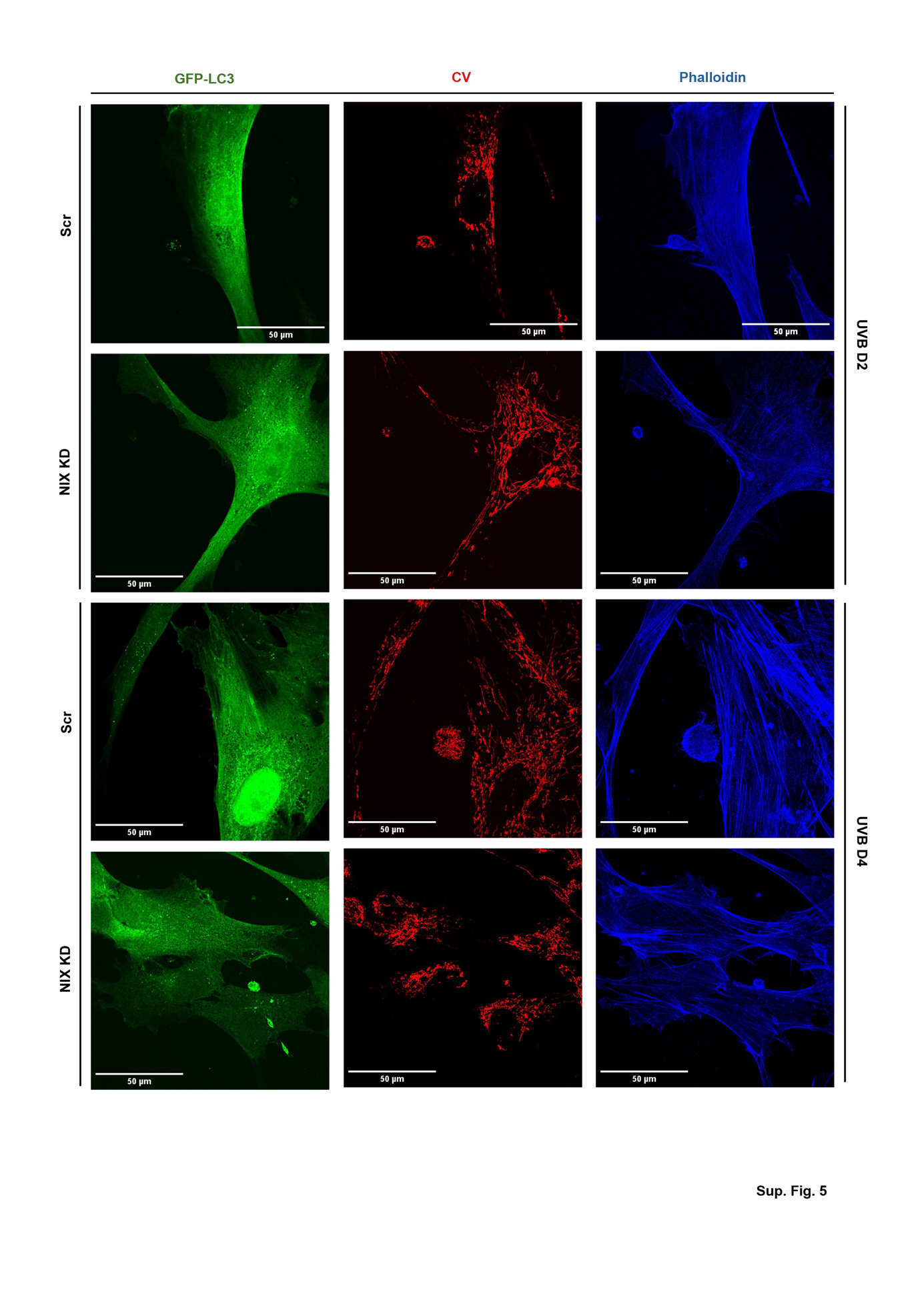
**

**Supplementary Figure 5: Release of EVs is increased in response to impairment of mitophagy.** HDFs expressing GFP-LC3 transduced with lentiviral vectors carrying NIX or scrambled shRNAs were irradiated for 2 and 4 days, stained with complex V antibody and phalloidin blue and observed by confocal microscopy to evaluate the release of EVs. Images depict separated channels of figure 6 A.
