# Supplementary material for: Elimination of damaged mitochondria during UVB‐induced senescence is orchestrated by NIX‐dependent mitophagy
Source: Aging Cell. 2024 May 17;23(8):e14186. doi: 10.1111/acel.14186 (PMC11320349; doi:10.1111/acel.14186)
Supplement: Supplementary file 2 — Video S1. [file ACEL-23-e14186-s001.zip › acel14186-sup-0002-VideoCaption.docx]

Video S1: Damaged mitochondria are eliminated by mitophagy. HDF expressing G

FP-LC3 and mRFP were irradiated for 2 days (UVB D2) and processed for live-cell microscopy. Mitophagy was monitored by engulfment of mitochondria (red) by autophagosomes (green).
